# Landscape of gene expression variation of natural isolates of *Cryptococcus neoformans* in response to biologically relevant stresses

**DOI:** 10.1099/mgen.0.000319

**Published:** 2019-12-20

**Authors:** Chen-Hsin Yu, Yuan Chen, Christopher A. Desjardins, Jennifer L. Tenor, Dena L. Toffaletti, Charles Giamberardino, Anastasia Litvintseva, John R. Perfect, Christina A. Cuomo

**Affiliations:** ^1^​ Division of Infectious Diseases, Department of Medicine, Duke University School of Medicine, Durham, NC 27710, USA; ^2^​ Broad Institute of MIT and Harvard, Cambridge, MA 02142, USA; ^3^​ Mycotic Diseases Branch, National Center for Emerging and Zoonotic Infectious Diseases, Centers for Disease Control and Prevention, Atlanta, GA 30329, USA

**Keywords:** *Cryptococcus*, RNA-Seq, cerebrospinal fluid (CSF), macrophage

## Abstract

*Cryptococcus neoformans* is an opportunistic fungal pathogen that at its peak epidemic levels caused an estimated million cases of cryptococcal meningitis per year worldwide. This species can grow in diverse environmental (trees, soil and bird excreta) and host niches (intracellular microenvironments of phagocytes and free-living in host tissues). The genetic basic for adaptation to these different conditions is not well characterized, as most experimental work has relied on a single reference strain of *C. neoformans*. To identify genes important for yeast infection and disease progression, we profiled the gene expression of seven *C. neoformans* isolates grown in five representative *in vitro* environmental and *in vivo* conditions. We characterized gene expression differences using RNA-Seq (RNA sequencing), comparing clinical and environmental isolates from two of the major lineages of this species, VNI and VNBI. These comparisons highlighted genes showing lineage-specific expression that are enriched in subtelomeric regions and in lineage-specific gene clusters. By contrast, we find few expression differences between clinical and environmental isolates from the same lineage. Gene expression specific to *in vivo* stages reflects available nutrients and stresses, with an increase in fungal metabolism within macrophages, and an induction of ribosomal and heat-shock gene expression within the subarachnoid space. This study provides the widest view to date of the transcriptome variation of *C. neoformans* across natural isolates, and provides insights into genes important for *in vitro* and *in vivo* growth stages.

## Data Summary

All RNA-Seq (RNA sequencing) data for this project are available in the National Center for Biotechnology Information (NCBI) Sequence Read Archive (SRA) linked to BioProject PRJNA167480 (https://www.ncbi.nlm.nih.gov/bioproject/?term= PRJNA167480). The processed gene expression data are available in the Gene Expression Omnibus (GEO) database under accession number GSE136879 and in Table S1(a, b) (available with the online version of this article).

**Impact Statement**

*Cryptococcus neoformans* is an environmentally acquired fungal pathogen that causes a large global impact of mortality and morbidity due to meningoencephalitis in immunocompromised patients. Most previous studies have examined clonally related laboratory strains from a single lineage. Here, we survey gene expression profiles across six isolates from the VNI and VNB lineages, including three closely related pairs of clinical and environmental isolates grown in five *in vitro* or *in vivo* conditions. Variation in gene expression is largely driven by genetic distance; differences between VNI and VNB on pigeon guano may reflect the natural niche of VNI on this nitrogen source. Gene expression *in vivo* highlights the metabolic and stress adaptive capabilities of this species. Expression profiles shared across diverse isolates help highlight the core transcriptional responses for this species. Furthermore, variation between isolates may suggest how some isolates have become better adapted to cause mammalian disease.

## Introduction

*Cryptococcus neoformans* can infect the central nervous system (CNS) resulting in life-threatening cryptococcal meningoencephalitis. Cryptococcal disease has had an enormous impact on worldwide health-care systems in the last three decades during the AIDS pandemic. This reached a peak of over one million patients per year resulting in over 600 000 deaths [1]. In the recent era of widespread use of effective antiretroviral therapies, the incidence has been reduced, but still remains high at over 220 000 new cases per year, with mortality rates approaching 50 % [2]. Understanding how *C. neoformans* grows in the host and causes disease will be paramount in its control.

*C. neoformans* can grow in a variety of environments. From its natural environmental niches in trees and bird guano, to its association with insects, amoebae and mammals, the environmental stresses encountered by *C. neoformans* are complex [3–6]. In the mammalian host, there are at least five stages of infection (initiation, dormancy, reactivation, dissemination and proliferation) that require efficient yeast adaptation to each environment [7–9]. Growth across these infection stages requires reprogramming of gene expression to respond to different nutrient availability and stressors. Hallmarks of the transcriptional responses of *C. neoformans* to the intracellular environment within phagocytic cells include metabolic shifts and activation of stress responses similar to those of other pathogenic fungi [10, 11]. However, we have only a limited understanding of other key stages of the infection life cycle to date. For instance, the transcriptional profile at the site of human infection in cerebrospinal fluid (CSF) has been analysed only relative to growth in rich media [12].

Genetic studies have established that *C. neoformans* var. *grubii* comprises four major lineages (VNI, VNII, VNBI and VNBII) that differ in global distribution, environmental niche and the level of genetic variation. Yet, most prior work examining gene expression has utilized a closely related set of *C. neoformans* strain backgrounds. The most commonly studied laboratory strain (H99) and a pair of strains congenic with H99 representing each of the two mating types (KN99ɑ and KN99a) fall within the globally represented VNI *C. neoformans* lineage. However, there is considerable genetic and phenotypic variation across isolates in each lineage, even within VNI [13, 14]. Isolates from the less commonly observed global lineage, VNII [13, 15], and lineages predominantly found in southern Africa, VNBI and VNBII [13, 16], have not been extensively studied to date. Notably, the environmental reservoir differs between the global VNI and VNII lineages, which can be frequently isolated from pigeon guano, and the more restricted VNB lineage found in association with mopane and other trees [17].

We undertook this study to compare the transcriptional variation of the *C. neoformans* lineages of both clinical and environmental isolates grown in key *in vitro* and *in vivo* conditions to help define patterns of gene expression variation across this species. The seven isolates selected include two closely related clinical and environmental isolate pairs from the VNI lineage representing different multilocus sequence typing genotypes, one pair from the VNBI lineage, and the standard laboratory strain H99 (Table 1). The two VNI environmental isolates, A1-35-8 and A5-35-17, were collected from pigeon guano and were previously shown to be avirulent in a murine intranasal model, whereas both the H99 and C23 VNI clinical isolates have been shown to be highly virulent in this model [18]. A separate study found that the CHC193 VNI clinical isolate was avirulent in this model, in contrast to the high virulence observed for H99 [19]. The pair of VNBI isolates has not been evaluated in the murine model; the clinical isolate was obtained from a CSF sample and the environmental isolate was collected from a mopane tree. We profiled the gene expression of these isolates in three *in vitro* conditions: growth on rich medium, on limited artificial medium and on PG medium. We also examined gene expression during interactions with macrophages as an intracellular host challenge and during *in vivo* infection producing disease within the CNS in an animal model. By comparing these conditions, we have identified genes that were specifically regulated in response to each condition and characterized the major patterns of expression variation between isolates representing the VNI and VNB lineages, as well as the subtler expression changes differentiating the closely related clinical and environmental isolates from the same lineage.

**Table 1.** Clinical and environmental isolates used in this study

Isolate	Group	Mating type	Origin	Source	Description
CHC193	VNIa	*MAT* alpha	China	Clinic	CSF (HIV−)
A5-35-17	VNIa	*MAT* alpha	USA	Environment	Pigeon guano
A1-35-8	VNIb	*MAT* alpha	USA	Environment	Pigeon guano
C23	VNIb	*MAT* alpha	USA	Clinic	Bronchial wash (HIV−)
H99	VNIb	*MAT* alpha	USA	Clinic	CSF (Hodgkin's lymphoma)
Bt85	VNBI	*MAT* a	Botswana	Clinic	CSF (HIV+)
Tu401-1	VNBI	*MAT* alpha	Botswana	Environment	Mopane tree

HIV, Human immunodeficiency virus.

## Methods

### Growth conditions

Descriptions of the *C. neoformans* isolates used in this study are listed in Table 1. The isolates were grown under different culture conditions for 24 h, including growth in yeast extract-peptone-dextrose (YPD), starvation-low-iron medium (SLIM) and pigeon guano (PG) media. Each isolate was grown on YPD agar for 2–3 days at 30 °C. Single colonies were inoculated into YPD broth and then incubated at 37 °C for 24 h. For the SLIM growth conditions, the isolates were grown in culture medium comprising yeast nitrogen base without copper and iron (YNB-CuSO_4_-FeCl_3_), 0.5 g glucose, 0.17 g ammonium sulfate and 0.1 M MOPS buffer. Immediately, before yeast inoculation, the medium was supplied with 0.15 mM tBOOT (tert-butylhydroperoxide), which penetrates the cells and produces reactive oxidative species that may mimic *in vivo* oxidative stress. The PG medium was prepared according to Nielsen *et al*. [20]. Briefly, 12 g pigeon guano was collected around Durham, NC, USA, resuspended in 1 l distilled water and autoclaved for 30 min. Next, the sterilized pigeon guano suspension was filtered through cheese cloth, autoclaved again for 15 min and stored frozen at −80 °C until use.

Macrophage-like murine cells (J774) were grown for 48 h, followed by washing and incubating with Dulbecco’s modified Eagle's medium (DMEM), and then incubated with DMEM plus 150 ng phorbol myristate acetate ml^−1^ in DMSO for 2 h. Yeast cells were grown in YPD, washed twice in PBS and opsonized with 10 µg murine mAb 18B7 ml^−1^ for 1 h. Then, 10^6^ yeast cells were added to 10^6^ activated J774 cells for 2 h. The unbound *Cryptococcus* cells were removed by washing with PBS. Macrophages loaded with intracellular yeast cells were incubated for 24 h at 30 °C in DMEM and then lysed with 0.05 % SDS in PBS (MP condition). Yeast cells were isolated by centrifugation and frozen at −80 °C.

Yeast cells were washed in PBS and then 0.3 ml of a 3.3×10^9^ c.f.u. ml^−1^ suspension of yeast cells from each isolate were intracisternally inoculated into sedated individual corticosteroid-treated 2–3 kg New Zealand white rabbits (three rabbits per isolate). After 24 h, CSF was withdrawn from the cisternal space of the three sedated rabbits for each isolate, pooled and centrifuged to pellet cells then frozen at −80 °C (CSF condition). Experiments were approved and monitored by the Duke Institutional Animal Care and Use Committee.

### RNA extraction, sequencing and differential expression analysis

To process samples for RNA extraction, the frozen yeast cell pellets were lyophilized before RNA isolation. These lyophilized samples were treated with TRIzol (Life Technologies) and total RNA was extracted using the an RNeasy mini kit (Qiagen), according to the manufacturer’s instructions. Library preparation was carried out by shearing the RNA and adapting mRNA for Illumina sequencing using the TruSeq RNA library prep kit (Illumina). The cDNA libraries were sequenced on a HiSeq 2000 (Illumina) to generate 101 bp paired-end sequencing reads. After quality filtering and adaptor trimming by Cutadapt (v 1.12) [21], RNA-Seq (RNA sequencing) reads (Table S1b) were aligned to the *C. neoformans* var. *grubii* H99 (CNA3, GCA_000149245.3) [22] or Bt85 (Cryp_neof_Bt85_V1, GCA_002215835.1) [16] genome using star (v 2.5.3a) [23] for RNA-Seq mapping, following ‘Alternate Protocol 7’ for star alignment with transcript coordinate output and with rsem (v1.2.31) quantification [24]. First, index files were created using the genome fastas and GFF files of gene coordinates, excluding non-coding RNAs and mitochondrial genes. Next, reads were mapped and transcript counts output (parameter --quantMode TranscriptomeSAM). After trimming and mapping to H99, approximately 21–62 million total paired aligned reads were recovered from each of these RNA-Seq libraries. The samples from the MP condition had fewer total reads, since lower amounts of RNA were isolated from the yeast cells co-incubated with macrophages. The number of yeast cells isolated from the CSF of rabbits is limiting; therefore, we pooled the yeast cells from two to three rabbits per isolate. The read counts for each gene were then estimated with rsem (v1.2.31) [25]. Subsequently, the raw count data was converted to c.p.m. and then normalized by adjusting with the effective library size via the calcNormFactors function implemented in the R package edgeR (v. 3.16.5) [26]. Differentially expressed genes (DEGs) were calculated between sets of samples listed in Table S2, using the negative binomial generalized linear models with quasi-likelihood tests (glmQLFit and glmQLFTest functions in the edgeR package) with False Discovery Rate (FDR) adjusted *P* values of less than 0.01. For the identification of lineage-specific DEGs and clinical-environmental DEGs (Table S2), the exact test was performed by using the exactTest function in edgeR. FDR *P* values of less than 0.05 were considered significant . The common set of lineage-specific DEGs corresponds to the intersection of the sets identified comparing VNI to VNB isolates for each condition (Table S3a). Multi-dimensional scaling (MDS) plots, hierarchical clustering and heat maps were constructed using the R packages edgeR and gplots (v.2.14.2). To identify CSF-specific expression patterns, DEGs in CSF compared to each other condition with the exception of PG medium were identified; the expression levels of the resulting 4922 genes were then clustered using k-means clustering with the R function kmeans (v. 3.3). Several values of k (*k*=4–8) were performed for k-means clustering. Venn diagrams were generated using the web tool Venn Diagrams (http://bioinformatics.psb.ugent.be/webtools/Venn/).

### Functional annotation of DEGs

To classify the DEGs, we carried out enrichment tests of functional annotation categories for sets of condition-dependent DEGs based on the known H99 gene annotation, *Saccharomyces cerevisiae* gene orthologs (from FungiDB release 45), Gene Ontology (GO), kegg and conserved Pfam domains [22]. In addition, we summarized and curated their known and predicted functions based on the limited information from FungiDB [27] or from previous studies (cited literature). For the detection of enriched functional pathways in each DEG gene set, pathway enrichment analysis was conducted for the GO terms and the kegg pathway [28] for *C. neoformans* H99. Terms containing *P* values ≤0.05 (from Fisher's exact test) were considered to be statistically enriched. The orthologs from each lineage were identified in a previous study based on blastp pairwise matches with *E* value <1.0e−5 [16] across 28 genomes of the VNI lineage and 9 genomes of the VNB lineages.

### Genomic data and variant identification

The genome sequences and SNPs for the seven isolates used for RNA-Seq analysis were obtained from previous studies [13, 16]. VCFtools (v 0.1.13) was used to parse the VCF files to filter and select subsets of variants [29].

## Results

### Genetic characterization of selected *C. neoformans* isolates

To evaluate how the genetic background and isolation source of *C. neoformans* impacts transcriptional responses, we selected seven isolates, including the reference strain H99 and three closely related pairs of clinical and environmental natural isolates (Table 1). A1-35-8 and C23 are environmental and clinical isolates, respectively, of VNI lineage subgroup b or VNIb, which are closely related to the reference strain H99 [13, 15]. A5-35-17 and CHC193 are environmental and clinical isolates, respectively, of VNI lineage subgroup a or VNIa [13, 15, 19]. Bt85 and Tu401-1 are closely related clinical and environmental isolates, respectively, of the VNBI lineage [17, 30]. Comparing SNP variants identified from whole-genome sequences [13] confirmed the close relationship of the two VNI pairs. A1-35-8 and C23 differ by only 341 SNPs; both isolates are also very similar to H99, with C23 differing at 388 SNPs and A1-35-8 differing at 505 SNPs. The A5-35-17 and CHC193 genomes show slightly higher pairwise variation, with 749 SNPs. In contrast, there are 73 294 SNPs that differ between the two VNBI isolates, Bt85 and Tu401-1, consistent with the higher average nucleotide diversity in VNB compared to VNI isolates [13].

### Genetic background impacts gene expression differences

To gain insight into gene expression differences between VNI and VNB lineages, and between related clinical and environmental isolates, we compared the expression profiles of these seven isolates in five conditions (Table S1b). We selected two artificial media, YPD, which provides nutrients to support optimal yeast growth, and SLIM, which contains only trace amounts of iron and copper [31] and an added oxidative stress molecule (tert-butylhydroperoxide). We also grew yeast in a PG medium that mimics the predominant environmental niche of VNI isolates and provides unique nutrients [20]. To compare and characterize expression profiles during certain pathogenic stages of cryptococcal meningitis, we examined these isolates in two *in vivo* conditions, survival within macrophages (MP condition) and in rabbit CSF. We conducted two biological replicates for each isolate in each condition, except for the rabbit CSF samples in which samples from multiple rabbits were pooled for analysis. Total RNA was isolated from each condition, which was selected for mRNA and adapted for strand-specific Illumina sequencing (see Methods). Reads were mapped to the H99 reference genome, which has deep RNA-Seq and other evidence for gene structure prediction [13]. The number of reads varied depending on the condition. For samples from SLIM, PG, YPD and CSF conditions, approximately 20–40 million reads were mapped; 2–10 million reads were mapped for the MP samples. One sample (H99 in the CSF condition) was excluded from analysis due to the relatively small number of mapped reads (102 697). After estimating the count of reads aligned to each gene, we retained a total of 6821 genes detected (read counts >1 c.p.m. in at least two samples) under our experimental conditions out of the 6962 genes that have been predicted for H99 [22] (Table S1a).

**Fig. 1.**
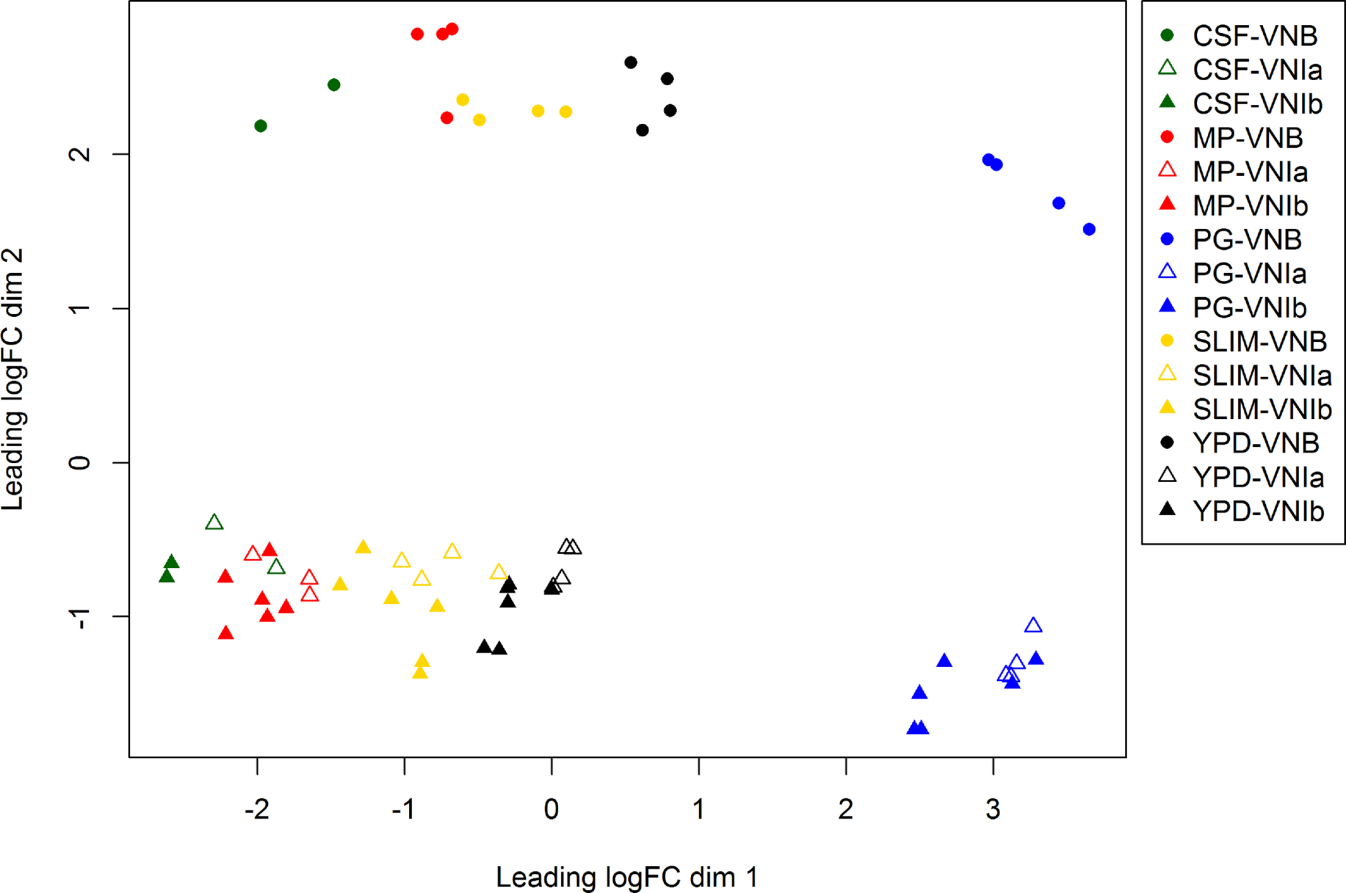
MDS plot of the RNA-Seq data sets for all samples. The distances in the MDS plot correspond to log2-fold changes between the gene expression measurements (normalized c.p.m.) of each pair of RNA samples. The leading logFC (log-fold-change) dimension 1 (horizontal axis) separates the different conditions, while the dimension 2 (vertical axis) separates VNBI samples from VNI samples. This plot exhibits the clear separation between VNI isolates, including VNIa (open triangles) and VNIb (filled triangles) and VNBI isolates (circles). This plot also highlights the variation across different conditions, including CSF (green), MP (red), PG (blue), SLIM (yellow) and YPD (black).

Prior to analysis of differential expression, we first examined the relationship of the sampled conditions and isolates. Comparing the samples, the highest correlation was between isolates in the same experimental condition; comparing between conditions, the two *in vivo* conditions (CSF and MP) appeared most similar in their expression patterns (Fig. S1). Most of the replicate samples were highly correlated, establishing the high reproducibility of the expression profiles of these samples. We also examined how samples clustered in a MDS plot (Fig. 1). Notably, samples from PG medium appear most different compared with all other conditions, suggesting a substantially different transcriptional response in this condition. Across all samples, the first dimension separated the different conditions and the second dimension separated VNB samples from VNI samples. Differences between the two VNI sub-lineages are relatively small, likely corresponding to their smaller degree of genetic variation than between VNI and VNB. These results established that in addition to the condition examined, the VNI versus VNBI lineage group origin is a major factor in gene expression variation.

To compare the response to different stress conditions across these *C. neoformans* isolates, we also examined the expression differences within each condition. Here, MDS plots for each condition separated the VNI from VNB isolates, and in three conditions (CSF, MP and YPD) also separated VNIa from VNIb; the isolates did not cluster based on environmental or clinical origin (Fig. S2). In most conditions, VNI and VNB samples are well separated along the first dimension and the VNI sub-lineage pairs separated along the second dimension. In the SLIM condition, the VNB clinical and environmental isolates appear separated along dimension 2 (Fig. S2); several genes involved in melanin biosynthesis are expressed at lower levels in clinical isolates compared to environmental isolates, including *HOG1* (CNAG_01523), *LAC1* (CNAG_03465), *MBS1* (CNAG_07464) and *BZP4* (CNAG_03346) (Table S4a). Loss of function mutations in *BZP4* were previously associated with clinical isolates compared to environmental in VNB, although loss of function mutations were identified in only four isolates [13]. The finding that expression of genes involved in melanin biosynthesis also varies between the clinical and environmental VNB isolates tested here may help explain the higher melanization (*P*<0.00047) and resistance to oxidative stress (*P*<0.00098) reported for VNBI environmental compared to clinical isolates, including for this pair of VNB isolates [13]. The samples within each sub-lineage appear more similar for YPD compared to other conditions, suggesting less impact of inter-lineage variation on growth in rich media. Overall this suggests a strong relationship between genetic distance and gene expression.

### Characterization of lineage-specific DEGs during *in vivo* growth

To evaluate lineage-specific gene expression, we identified significantly DEGs between the VNI and VNB lineages in each condition (Tables S5a–e and S6, Fig. S3). In most conditions, approximately 1000–1300 DEGs were identified, except CSF where only ~270 DEGs were found. The lower number of DEGs for the CSF condition might be due to the smaller sample number for this condition. We performed functional annotation analyses and literature searches for these lineage-specific DEGs from each condition. Using functional and pathway analyses, we found several DEGs that are involved in fungal virulence or yeast survival in the host (Table S6) [32–34]. Several lineage-specific DEGs play a role in resistance to oxidative damage, a critical component of host immune cell defences. Two 5-oxoprolinase genes, CNAG_06872 and CNAG_06873, were identified as VNI up-regulated in both CSF and MP conditions, compared to VNB isolates, and a third 5-oxoprolinase gene, CNAG_02464, is significantly up-regulated in macrophages for VNI isolates. 5-Oxyprolinases are involved in glutathione biosynthesis, and play roles in fungal development and virulence [35]. In addition, two ferric-chelate reductase genes were differentially regulated in the CSF and MP conditions; *FRE3* (CNAG_06524) is up-regulated in VNI, while *FRE6* (CNAG_06976) is down-regulated in VNI. Ferric reductases are critical for *C. neoformans* survival during iron-dependent growth and for causing disease in the host. The differential expression of these genes between two lineages might affect the oxidative stress resistance, the acquisition and reduction of iron, and virulence of these isolates in the host [31, 36, 37].

In addition, several lineage-specific DEGs from *in vivo* conditions were associated with other known virulence factors of *C. neoformans*. Many lineage-specific DEGs involved in capsule production, the most dominant virulence associated factor, were regulated during MP interaction (Table S6). Other genes up-regulated in both *in vivo* conditions in VNI include: *APP1* that encodes a secreted extracellular antiphagocytic protein, which inhibits the attachment and ingestion of yeast cells by alveolar macrophages; *CXD3*, a carboxypeptidase D correlated with capsule size [38]; and *SRX1*, a sulfiredoxin required for cells to counteract peroxide stress and virulence of *C. neoformans* in mouse infection models [39]. VNI lineage-specific DEGs down-regulated in CSF include *OCH1*, an alpha-1,6-mannosyltransferase involved in the *N*-glycan processing in the glycosylation pathway of *C. neoformans* [40]. Additionally, two ATP-binding cassette transporter genes, *PDR5* from both *in vivo* conditions and *PDR5-2* from the MP condition, were VNI down-regulated DEGs. Thus, within certain lineages there is specific network wiring of genes involved in host interaction. Furthermore, the function of approximately 50 % of lineage-specific DEGs from each condition remain undefined (Fig. S4), which will require functional characterization to understand differences in expression between lineages.

### Identification of a common set of lineage-specific DEGs across all conditions

A total of 94 DEGs are differentially expressed between VNI and VNB isolates in all five conditions based on mapping to the H99 genome. Of these, 88 genes are VNI up-regulated and 6 are down-regulated (Table S3a). To more closely examine genes specific to the VNB lineage, reads were also mapped to the VNBI (Bt85) gene set [16] and analysed for differential expression. From Bt85 reference mapping, 46 lineage-specific DEGs were found in the common set of all conditions (FDR *P* value<0.05) (Table S3b). Of these 46 DEGs, 14 genes are up-regulated in VNI isolates and 32 genes are down-regulated (corresponding to higher gene expression in VNBI isolates). By mapping orthologs between the Bt85 and H99 genomes, we combined the lineage-specific DEGs for a total of 114 lineage-specific DEGs (Table S3c). These genes are involved in transmembrane transport (drug transport, allantoate permease, maltose permease, ATP-binding cassette transport and spermine transport), protein folding (peptidylprolyl isomerase), regulators of transcription, oxidation–reduction processes (sulfonate dioxygenase, ferric-chelate reductase and monooxygenase), amidohydrolase and haloacid dehalogenase. These functions that are expressed across conditions in only one lineage suggest core regulation that has differed as the two lineages evolved separately in association with different environmental niches.

### Common sets of lineage-specific DEGs from each condition showed both loss of genes and their location enrichment in subtelomeric regions

The majority of the DEGs that were up-regulated in VNI compared to VNB are present in VNI but absent in VNB. In examining the properties of lineage-specific DEGs mapping to the H99 genome, we found that most of these genes present in one lineage but absent in the other lineage were clustered in five genomic regions, including regions on chromosomes 1, 4, 5, 7 and 8. The largest cluster, located at chromosome 7 coordinates 384 496 to 394 513 in H99 and noted in a previous comparison of these genomes [16], includes five genes: an acetyltransferase, an amidohydrolase and an allantoate permease. To determine whether this observation was specific to Bt85 or whether these genes were also missing in other VNB isolates, we compared eight other VNB genomes and found that most of these genes absent in Bt85 were also missing in other VNB isolates (Table S3b).

**Fig. 2.**
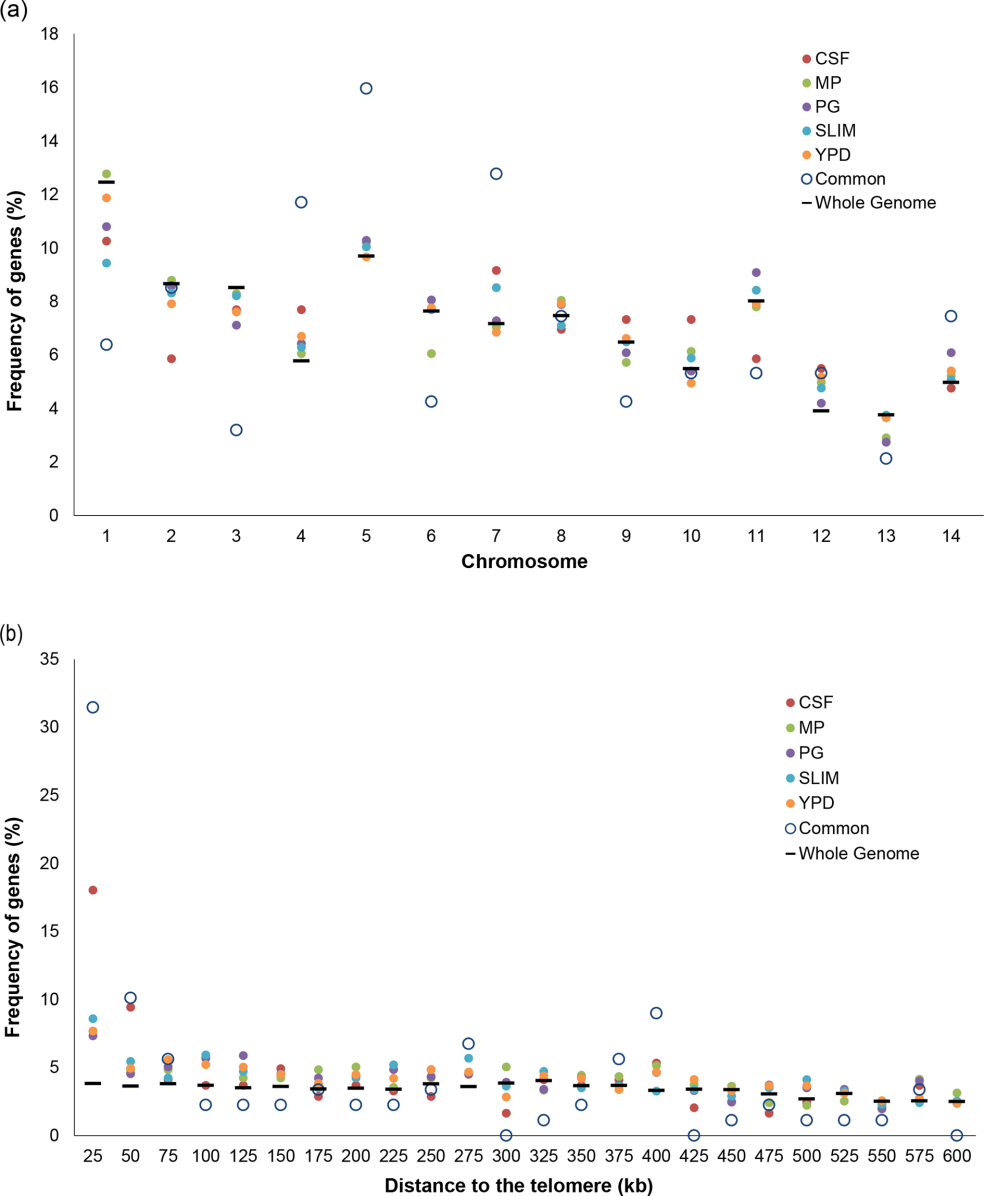
Chromosomal location enrichment of DEGs between VNI and VNB in all conditions. (a) Frequency of lineage DEGs compared to all genes for each chromosome of H99. The common category is the intersection of lineage-specific DEGs across all five conditions (94 genes). The whole-genome category includes all genes in the H99 genome (6962 genes). (b) Frequency of lineage-specific DEGs with the distance relative to the closest telomere.

To further characterize the basis of lineage-specific gene expression, we mapped the chromosomal location of all lineage-specific DEGs and examined their divergence between the two lineages. First, we tested for chromosomal enrichment of lineage-specific DEGs. Comparing between the lineage-specific DEGs and all genes in the whole genome, we found that the common set of DEGs are significantly enriched on chromosomes 4, 5 and 7 (chi-square *P* values=0.013, 0.039 and 0.034, respectively) (Fig. 2a). Next, we examined the chromosomal neighbourhood of these DEGs by measuring relative distance from chromosome ends of the H99 genome and found that lineage-specific DEGs are significantly enriched in subtelomeric regions at a distance less than 25 kb from the end of the chromosome (chi-square *P* value <0.0001) (Fig. 2b). Genes in subtelomeric regions have been shown to have higher selection pressure between lineages in population genomic data [13]. Four of the five VNI specific gene clusters are located in the subtelomeric regions, except for the gene cluster on chromosome 7 (Table S3a). These results support that lineage-specific expression is influenced by lineage-specific genes, many of which are associated with subtelomeric regions.

### PG-specific DEGs identify substantial environmental control to gene expression

As noted above, growth in pigeon guano displayed the most distinct gene expression profiles compared to other conditions. We identified 1068 and 1180 DEGs that are specifically up-regulated or down-regulated in the PG medium condition across all isolates, respectively (Table S7a, b). Enriched kegg pathways of PG-specific up-regulated genes included those in fatty acid degradation, valine, leucine and isoleucine degradation, and carbon fixation pathways (Table S7c). These up-regulated DEGs are also enriched for glutathione metabolic processing, protein modification, response to oxidative stress (cytochrome c peroxidase), peroxisome fission and organization, and ubiquitin-dependent protein catabolic processes. Furthermore, seven PG-specific up-regulated DEGs were involved in nitrogen compound metabolic processing (Table S7d). *URE7* and *URE1*, genes encoding a urease accessory protein and urease, respectively, were up-regulated suggesting that this yeast may be utilizing urea present in the pigeon guano. Urease has been previously found to be an important virulence factor for *C. neoformans* [20, 41]. Ammonium and glutamine are the preferred nitrogen sources of most fungi, but in the absence of these sources, less easily assimilated nitrogen sources may also serve as sources of nitrogen [42–44]. However, two genes of glutamine metabolism, glutamine synthetase (CNAG_00678) and glutamate synthase (CNAG_04862), were also up-regulated, suggesting that *C. neoformans* utilizes multiple pathways to acquire nitrogen. By contrast, the PG-specific down-regulated DEGs are enriched in aminobenzoate degradation, nucleotide metabolism, amino acid metabolism and phenylpropanoid pathways.

Furthermore, we also identified several lineage-specific DEGs linked to nitrogen source usage, which may benefit the growth of VNI lineage isolates in pigeon guano, comparing to VNB isolates. Two VNI up-regulated DEGs from *in vivo* and PG medium conditions, CNAG_06652 and CNAG_06875, are homologous to the *S. cerevisiae* allantoate permease (*DAL5*), which is involved in uptake of uric acid products and plays a role in the utilization of certain dipeptides as a nitrogen source [45, 46]. Another VNI up-regulated DEG in *in vivo* and PG medium conditions, CNAG_01161 shares sequence similarity with Dcg1 (a region of 24 % identity covering 76 % of the protein), which is potentially involved in nitrogen compound metabolic processing in *S. cerevisiae* [47, 48]. Four additional lineage-specific DEGs genes are associated with urea metabolic processes. These genes include an allantoate transporter, CNAG_06561 (similar to *DAL5*), and a urea carboxylase/amidolyase, CNAG_07944 (*DUR1*/*DUR2*), which is involved in urea carboxylase and allophanate hydrolase activity [49, 50]. The cryptococcal urease gene, CNAG_05540 (*URE1*), and a urease accessory gene, CNAG_02057 (*URE6*), have been shown to be essential for the uric acid degradation pathway, and *URE1* plays an important role in virulence [41, 43, 51, 52]. By contrast, CNAG_06694 (*URO2*) was identified as a VNI down-regulated DEG from *in vivo* and PG medium conditions, and encodes a hydroxyisourate hydrolase that is involved in the uric acid degradation pathway [53, 54]. Overall, the regulation of urease activity may be critical for *C. neoformans* to break down nitrogen resources present in pigeon guano. The results suggest that these DEGs might provide an advantage for VNI isolates in their survival within pigeon guano compared to VNBI isolates, as it is likely beneficial for *C. neoformans* to uptake and utilize uric acid in bird droppings as a nitrogen source [41, 43, 54]. In addition, *C. neoformans* can utilize urease to help invade the CNS via the blood–brain barrier and cause life-threatening meningoencephalitis [55–57].

### Identification of DEGs during CNS growth

Few fungi can infect the CNS. *C. neoformans* can invade, survive and proliferate in the CSF of the CNS. Identifying DEGs associated with this specific host condition and characterizing how expression varies across genetically diverse isolates is critical to our understanding of the genes and regulatory networks enabling pathogenesis of the CNS. The cortisone-treated rabbit model has been widely used for cryptococcal meningitis due to its chronic meningoencephalitis progress and its larger body size, which allows serial study of yeasts at the site of infection, compared to mice and rats [58, 59]. Here, we compared the gene expression profiles of isolates in CSF collected from the rabbit model of cryptococcal meningitis at an early stage of infection and identified DEGs specifically associated with the growth within the subarachnoid space (Table S8, Fig. S5). Pairwise comparisons across the different conditions revealed that the smallest numbers (~1400) of DEGs was found between CSF and MP conditions, with an enrichment for kegg and GO terms involved in diverse metabolism and stress responses, described below (Table 2). In contrast, approximately 4200 DEGs were identified between CSF and either PG or YPD media, as gene expression profiles of these two environmental conditions provide vastly different signals and, thus, vary substantially from the yeast’s experience within the subarachnoid space.

**Table 2.** Enriched GO slim terms and kegg pathways between CSF and MP conditions

kegg pathway name/GO term	kegg ID/GO ID	Gene count	*P* value	FDR *P* value*
**CSF vs MP up-regulated**				
Tryptophan metabolism	ec00380	27	3.83e−03	3.38e−01
Selenocompound metabolism	ec00450	14	6.90e−03	3.38e−01
Peptidoglycan biosynthesis	ec00550	2	1.39e−02	3.62e−01
DDT degradation†	ec00351	4	1.48e−02	3.62e−01
Protein folding	GO:0006457	11	1.89e−04	**6.42e−03**
tRNA metabolic process	GO:0006399	13	1.61e−02	2.73e−01
**CSF vs MP down-regulated**				
Fatty acid degradation	ec00071	17	1.21e−09	**1.46e−07**
Citrate cycle (TCA cycle)	ec00020	16	6.47e−09	**3.88e−07**
Valine, leucine and isoleucine degradation	ec00280	18	1.21e−08	**4.84e−07**
Geraniol degradation	ec00281	10	8.45e−08	**2.54e−06**
Carbon fixation pathways in prokaryotes	ec00720	18	2.24e−04	**5.38e−03**
Synthesis and degradation of ketone bodies	ec00072	6	2.04e−03	**3.90e−02**
Caprolactam degradation	ec00930	4	2.27e−03	**3.90e−02**
Fatty acid elongation	ec00062	12	4.17e−03	5.77e−02
Pyruvate metabolism	ec00620	8	4.32e−03	5.77e−02
Steroid biosynthesis	ec00100	3	5.80e−03	6.96e−02
α-Linolenic acid metabolism	ec00592	10	6.41e−03	6.99e−02
Glycolysis/gluconeogenesis	ec00010	18	7.58e−03	7.58e−02
Propanoate metabolism	ec00640	6	9.80e−03	9.04e−02
Primary bile acid biosynthesis	ec00120	16	2.22e−02	1.90e−01
Naphthalene degradation	ec00626	5	4.00e−02	2.97e−01
Starch and sucrose metabolism	ec00500	5	4.00e−02	2.97e−01
Benzoate degradation	ec00362	9	4.30e−02	2.97e−01
Generation of precursor metabolites and energy	GO:0006091	14	2.82e−05	1.21e−03
Cell wall organization or biogenesis	GO:0071554	11	1.66e−04	3.57e−03
Biological process	GO:0008150	311	2.81e−04	4.03e−03
Lipid metabolic process	GO:0006629	18	5.16e−03	5.55e−02
Vacuolar transport	GO:0007034	4	1.09e−02	9.35e−02
Response to stress	GO:0006950	26	1.62e−02	1.01e−01
Carbohydrate metabolic process	GO:0005975	21	1.64e−02	1.01e−01
Secondary metabolic process	GO:0019748	16	1.99e−02	1.07e−01
Reproduction	GO:0000003	9	4.79e−02	2.00e−01

*Values below an FDR value of 0.05 are shown in bold type.

†1,1,1-Trichloro-2,2-bis (4-chlorophenyl) ethane (DDT) degradation.

TCA, tricarboxylic acid.

**Fig. 3.**
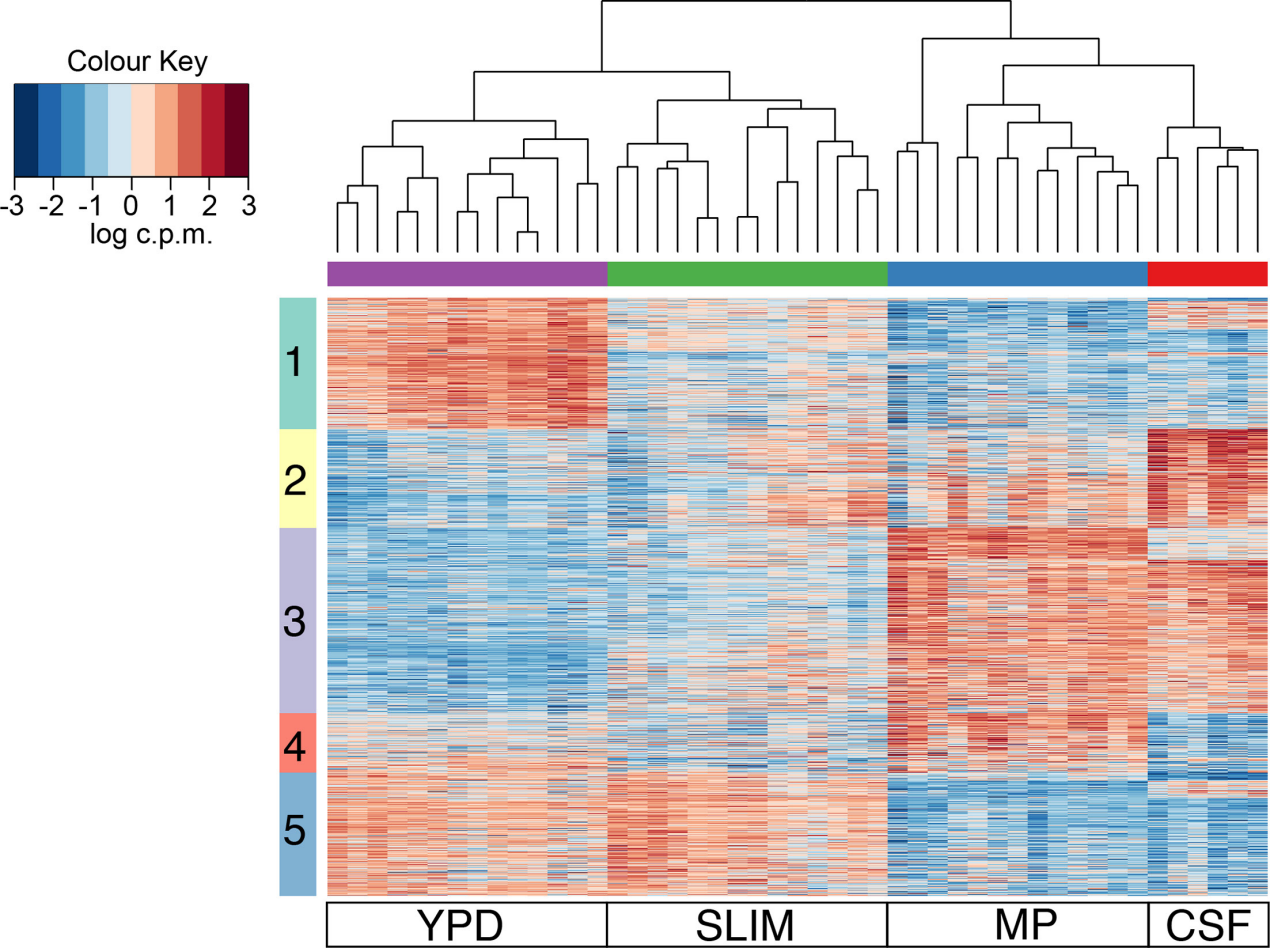
K-means clustering heatmap of all DEGs in CSF compared to MP, YPD and SLIM. Five major clusters were identified (*y*-axis); each row corresponds to a gene.

Next, we extracted the sets of DEGs identified in pairwise comparisons of the CSF condition with MP, SLIM and YPD. Comparison to PG medium was excluded as the expression differences were substantially larger compared to all other conditions. Comparison of expression levels was performed with k-means clustering for all samples, excluding PG medium; inspection of profiles at different *k* values identified five major clusters of expression profiles (Fig. 3, Table S9a–e). The five clusters can be defined by genes highly expressed in one or more conditions, including genes induced or repressed in one or both *in vivo* conditions. Then, GO and kegg pathway enrichment analysis on the DEGs in each cluster (Table S10) was performed. To estimate the clinical effect of these DEGs, we also categorized the virulence-associated gene mechanisms underlying each cluster.

#### Genes up-regulated during *in vitro* growth

Genes differentially regulated during growth in YPD or SLIM media were present in two clusters. Cluster 1 (1081 genes) includes genes with highest expression in YPD and lower expression in all other conditions. The most enriched pathways found in cluster 1 were related to aminoacyl-tRNA biosynthesis, pantothenate and coenzyme A (CoA) biosynthesis, and various amino acid metabolism. Cluster 5 (1017 genes) contains genes with increased expression in YPD, as well as SLIM, which were mainly involved in vesicle-mediated transport, cofactor metabolisms, carbohydrate metabolisms and lipid metabolism. Many genes in cluster 1 and cluster 5 were associated with cell wall and/or capsule formation, including several genes in the G protein-cAMP-PKA signalling pathway that promote mating and the production of virulence factors, melanin and capsule, for *C. neoformans* [60, 61]. Other genes, such as chitin deacetylases, are involved in cell wall structure and integrity or in capsule production (Table S9a, e; Supplementary Note). Several other genes are linked to virulence, including the laccase, *LAC2*, which is involved in melanin production (Supplementary Note).

#### ﻿Genes up-regulated in *in vivo* conditions

Genes up-regulated in both *in vivo* conditions and down-regulated in the *in vitro* conditions were present in cluster 3 (1522 genes). Gene functions related to lysine biosynthesis, inositol phosphate metabolism and inositol lipid modifications (including phosphatidylinositol phosphate kinase, phosphatidylinositol phosphate phosphatase, phosphatidylinositol phospholipase and inositol oxygenase), nitrogen metabolism, DNA recombination and repair, cell cycle, chromosome organization, and stress responses were enriched in this cluster. A total of 21 DEGs are involved in inositol transport or catabolism, such as myo-inositol transporters, inositol 2-dehydrogenases, inositol oxygenases, inositol/phosphatidylinositol kinases and phosphoinositide phospholipases (Table S9c; Supplementary Note). The human brain contains abundant inositol, and if glucose availability is low in the brain, *Cryptococcus* may utilize inositol as a carbon source. Notably, a set of ~120 virulence-associated genes were also identified within this cluster. Nearly half (47) of these genes are involved in capsule formation or cell wall integrity, including many capsule-associated proteins and genes involved in chitin synthesis and regulation (Supplementary Note). Other DEGs include two sodium efflux transporters, *ENA1* and *NHA1*, that cooperatively maintain the intracellular pH and homeostasis of several toxic cations [62], *OPT1*, which encodes an oligopeptide transporter required in Qsp1-dependent quorum sensing, which mediates autoregulatory signalling, modulates secreted protease activity and promotes cell wall function at high yeast cell densities [63, 64], and *PMC1*, a vacuolar calcium ATPase, which is critical for both the progression of pulmonary infection and brain colonization in a murine infection model [65, 66]. Stress response genes include *SRE1*, which encodes a sterol regulatory element-binding protein (SREBP), that is involved in oxygen sensing and sterol homeostasis, and is important for adaptation and growth of *C. neoformans* in the brain [67, 68], the Bck1-Mkk2-Mpk1 signalling pathway, which is activated in response to thermal stress [69, 70], and the pH-response transcription factor, *RIM101*. Interestingly, two uric acid catabolic genes, *URO2* and *URO3*, were also identified as up-regulated in *in vivo* conditions. Uric acid in human blood plasma serves as an antioxidant and is also released by necrotic mammalian cells as a danger signal to activate both innate and adaptive immune effectors [41].

#### Genes up-regulated in the CSF condition, down-regulated in the MP condition

Cluster 2 (814 genes) contains genes highly expressed in CSF (subarachnoid space) compared to all other conditions. Enriched functions are associated with ribosome biogenesis, tRNA metabolism, aminobenzoate degradation, selenocompound metabolism, polycyclic aromatic hydrocarbon degradation, and phenylpropanoid, flavonoid, stilbenoid, diarylheptanoid and gingerol biosynthesis, and amino acid metabolisms; most of these functions were also found in a direct comparison of each isolate in the CSF and MP conditions (Tables S9b and 2; Supplementary Note). Heat-shock protein associated pathways were also found in this cluster. Here, we identified several heat-shock related protein families (Hsp60/Hsp70/Hsp90/Hsp104). Previous studies have shown that the heat-shock family of *HSP70*, encoding ubiquitous chaperones, is involved in the cellular response to stress and associated with diverse pathological processes including programmed cell death [71]. For *C. neoformans*, Hsp70 serves as a stress-related transcriptional co-activator required for virulence [71]. Hsp104 in association with Hsp40 and Hsp70 helps in reactivation and aggregation of denatured protein [72]. Hsp90, which localizes at the cell wall, is involved in mediating thermotolerance. Hsp90 can also augment virulence factors, confer antifungal drug resistance and regulate capsule induction and maintenance [73]. These heat-shock proteins may also contribute to the repair or renaturation of damaged proteins during morphogenesis and various stress conditions [72, 74], including growth in CSF.

#### Genes up-regulated in the MP condition, down-regulated in the CSF condition

In contrast, cluster 4 (488 genes) includes genes with the highest expression in MP, but low expression in CSF, enriched for functions including fatty acid degradation, geraniol degradation, valine, leucine and isoleucine degradation, and glutathione and propanoate metabolism. The citrate cycle (tricarboxylic acid cycle) was also found in this cluster and in a pairwise comparison of MP to CSF (Tables S9d and 2).

As noted in a previous study, specific gene expression changes in response to the intracellular environments of macrophages or amoebae were discovered by comparing transcriptional profile of yeast cells internalized by murine macrophages or *Acanthamoeba castellanii* to cells grown in corresponding culture medium [10]. In total, 93 genes, including 70 up-regulated genes and 23 down-regulated genes, in both macrophages and amoebae were identified (Table S11a, b). To compare the expression of these genes across our isolates, we compared the transcriptional profiles in the intracellular environment of macrophages (MP) to culture medium (YPD). Of these 93 genes, 63 out of 70 MP up-regulated genes and 13 out of 23 MP down-regulated genes were validated in our data (MP vs YPD) (Table S11a, b). Most of these 63 validated up-regulated genes are involved in lipid metabolism, including acyl-CoA oxidase, acyl-CoA thioesterase, acyl-CoA dehydrogenase, acyl-CoA synthetase and acetyl-CoA acyltransferase. Other genes are linked to transport of sugars or amino acids, the glyoxylate cycle (isocitrate lyase and malate synthase A), nitrogen metabolism, 2-nitropropane dioxygenase and oxidative stress responses. While important for macrophage growth, the glyoxylate pathway is dispensable for *C. neoformans* within the subarachnoid space [75]. Furthermore, several studies have demonstrated that survival of *C. neoformans* within the host phagolysosome requires the ability to protect itself from oxidative stress. For instance, the gene encoding a cytochrome c peroxidase, which catalyses the degradation of hydrogen peroxide and can provide protection against host-derived reactive oxidative species [76, 77], is highly up-regulated in MP but is not essential for yeast virulence [77]. Another up-regulated gene, inositol oxygenase (CNAG_06623), is involved in conversion of inositol to glucuronic acid and controls the utilization of inositol as an energy source [78–80]. Our results more widely validate adaptation mechanisms of *C. neoformans* to the phagosomal microenvironment. However, as not all genes that respond to the macrophage interaction are required for virulence, there may be alternative pathways not identified by the transcriptomes important for survival *in vivo*.

### Differential expression between clinical and environmental pairs showed several virulence-associated regulations

Previous studies have shown differences in murine virulence between environmental and clinical isolates, even those with close genetic relationships [18, 81]. To identify differences that may contribute to the higher virulence of these clinical isolates, we investigated the expression differences between the pairs of closely related clinical and environmental isolates of the VNI lineages in CSF or MP condition (Tables S2 and S4b,c). Therefore, we could identify those limited but critical DEGs responsible for the differences in virulence between the genetically closely related isolates. The VNB samples were excluded from this analysis due to the limited number of isolates. From the functional and pathway analysis of these DEGs, some of these genes were found to be associated with virulence factors.

Examining gene expression of VNI clinical isolates compared to environmental isolates during CSF growth, 33 genes are up-regulated in clinical isolates and 9 genes are down-regulated. Half of these identified DEGs are of unknown function. Of the characterized DEGs, two genes induced in clinical isolates (CNAG_00373; CNAG_00895) and gene more highly expressed in environmental isolates (CNAG_06606) are associated with virulence factors. CNAG_00373 encodes the extracellular glucan 1,3-β-glucosidase, which participates in the metabolism of the most abundant fungal cell wall polysaccharide, β-glucan [82, 83]. Another up-regulated gene, *ZIP1* (CNAG_00895), encodes a zinc ion transporter that is involved in zinc uptake in *C. neoformans*. The targeted deletion of this gene showed attenuated virulence in a murine model and reduced survival within murine macrophages [84]. The down-regulated gene, *RHO104* (CNAG_06606), encodes a GTPase in Rho signalling pathways and the knockout strain of this gene has shown increased infectivity in the murine inhalation model [85], suggesting the lower expression levels observed in clinical isolates could provide an advantage *in vivo*.

Comparing VNI clinical to environmental isolates during macrophage interaction (MP) identified 205 genes, including some associated with fungal defence in macrophages and survival in the host. For instance, we found that a quinone oxidoreductase (CNAG_02933) is up-regulated in clinical isolates compared to the environmental isolates. Quinone reductase activity confers important antioxidant effects by reducing and detoxifying quinone molecules (Supplementary Note). An up-regulated siderophore-iron transporter, CNAG_00815, is involved in the titan-like cell formation in response to various stresses for *C. neoformans* [86]. CNAG_00815, which is up-regulated in clinical isolate, is associated with iron acquisition, melanin formation and cAMP signalling in *C. neoformans* [87]. These results suggest that despite the differences in virulence between the clinical and environmental isolates, the gene expression profiles are not grossly varied in these *in vivo* conditions. We interpret this as meaning that a few changes can lead to dramatic outcomes in terms of virulence. Having the genomes sequenced will enable further investigations to characterize the cause of the phenotypic differences between clinical and environmental isolates.

## Discussion

Studies of the molecular pathogenesis of *C. neoformans* have begun to take full advantage of whole-genome sequencing to characterize natural isolates [3, 13, 14, 16, 88]. This work built on prior studies that collected these isolates and used multilocus sequence typing to characterize the major divisions within this species and the relationship of clinical and environmental isolates [15]. While there are not well-established phenotypic differences between isolates from the VNI, VNII, and VNB lineages, phenotypic variation in virulence has been noted between clinical and environmental isolates [18]. Here, we characterized how related clinical and environmental isolates vary in gene expression across five conditions and provide a comprehensive view of transcriptional variation between these isolates.

Prior gene expression studies have characterized the response of *C. neoformans* to a wide variety of conditions. In a study of intracellular environment, yeasts inside macrophages showed up-regulation of transporters, oxidative stress responses along with autophagy, lipid metabolism and peroxisome functions [11]. In our comparison of MP to CSF conditions, we also observed a major up-regulation of lipid metabolism, which further studies have shown is important for full virulence in a mouse model [89]. Furthermore, *in vivo* gene expression studies used serial analysis of gene expression (SAGE) to quantitatively identify cryptococcal gene expression within the subarachnoid space of the rabbit [58] and uniquely identified the trehalose biosynthesis pathway as induced in the CSF. An RNA-Seq guided gene set [22] was used to study cryptococci taken directly from the human subarachnoid space, and captured the expression profiles at the CNS site of infection compared to growth in rich media [12]. Between the two patients examined in this prior study, many DEGs were found to be unique to each isolate but, importantly, the overall expression profile was generally similar between these two human cryptococcal isolates directly isolated from the host. Thus, while variation exists between the two isolates (VNI and VNII), there appeared to be a shared CSF-regulated cryptococcal transcriptome. Here, RNA-Seq of isolates from rabbit CSF also showed a common set of DEGs across divergent isolates. While this is still a small set of isolates, this begins to define aspects of the response in CSF that are common across isolates with different genetic background; future studies will be required to decipher which isolate specific DEGs could be important for virulence.

Our results showed that the major virulence factors, polysaccharide capsule and melanin production genes, are modulated under both *in vivo* and *in vitro* conditions. For instance, the cAMP-dependent signalling pathway is known to regulate capsule production, melanin formation, mating and virulence in *C. neoformans* [60]. A previous study proposed that *C. neoformans* might contain dual systems regulating the cAMP/PKA pathway in the maintenance of virulence attributes of the pathogen [90]. As for the biosynthesis mechanism of capsule formation, we found that *PKA1* and *PDE1* are highly expressed under *in vitro* conditions but in contrast, *PKA2* and *PDE2* are highly expressed under *in vivo* conditions. The close relationship of the MP and CSF expression profiles allowed more detailed definition of the gene expression profiles specific to each survival stage. This analysis has helped establish that during survival in CSF, yeast cells are focused on expression of transporters important for nutrient acquisition and appear to be in a protective mode against stress; these observations suggest that interruption of these protective functions has the potential to reduce or eliminate disease progression.

By utilizing diverse natural *C. neoformans* isolates, we also observed that gene expression variation between isolates from different lineages was much greater than between genetically related isolates from the clinic versus the environment. This highlights that further studies of gene expression profiles will need to carefully control for genetic background of the lineage and possibly even sub-lineage levels. Recently, in *Cryptococcus gattii*, an initial study described differences in expression across four major lineages [91]. While we have found that gene gain or loss in *C. neoformans* isolates explains a major part of the lineage-specific gene expression, further work is needed to map finer scale gene variation with the expression differences.

Differences detected in gene expression between isolates from VNI and VNB lineages could be related to the different environmental niches of these lineages. For instance, nitrogen is a major essential component for life, and fungi are known for their ability to use a range of catabolic enzymes to utilize many nitrogen sources, including uric acid, urea, creatinine, amino acids, pyrimidines and purines [41–44]. Bird excreta contain high concentrations of the insoluble heterocyclic compound uric acid that provides an easily assimilated nitrogen source. In this study, several genes involved in nitrogen catabolic enzymes were discovered to be differentially expressed between lineages. These regulated genes might provide a fitness advantage for VNI isolates to survive in pigeon guano compared to VNB isolates and support their known environmental niche.

In the set of *C. neoformans* isolates we examined, we were not able to detect dramatic differences of expression within the sub-lineage of the isolates. The low genome-wide variation of VNI isolates between the clinical and environmental pairs does not result in large-scale expression differences. Despite this similar gene expression profile between clinical and environmental VNI isolates, we have observed substantial differences in their ability to produce disease in mice [18]. Thus, the minor differences in gene expression between related environmental to clinical isolates might provide gene candidates to test for this virulence difference *in vivo*, including potentially the large subset of genes with currently unknown function, to pinpoint what changes enable this environmental pathogen to grow in a host. However, a more complete understanding of the differences in the virulence potential of this species will also require a wider comparison of genomic and epigenetic differences between isolates that vary in phenotype and further systematic testing of specific variants to be able to map the variants and expression changes associated with inherent virulence differences. While this study provides us with an initial picture of the variation in yeast transcriptional responses to a variety of environments, further characterization of how the population variation in gene expression programs affects infection and pathogenesis will be key to model and control responses for this fungus and other pathogens.

## Data bibliography

1. Cuomo, C. A., Litvintseva, A., Perfect, J. All RNA-Seq data for this project are available in the National Center for Biotechnology Information (NCBI) Sequence Read Archive (SRA) under BioProject accession number PRJNA167480 (https://www.ncbi.nlm.nih.gov/bioproject/?term= PRJNA167480https://www.ncbi.nlm.nih.gov/bioproject/?term= PRJNA167480) (2012).

2. Yu, C., Desjardins, C. A., Perfect, J. R., Cuomo, C. A. The processed gene expression data are available in the NCBI GEO under accession number GSE136879 (https://www.ncbi.nlm.nih.gov/geo/query/acc.cgi?acc=GSE136879) (2019).

## Supplementary Data

Supplementary material 1

Supplementary material 2
