# Supplementary material for: Landscape of gene expression variation of natural isolates of Cryptococcus neoformans in response to biologically relevant stresses
Source: Microb Genom. 2019 Dec 20;6(1):e000319. doi: 10.1099/mgen.0.000319 (PMC7067042; doi:10.1099/mgen.0.000319)
Supplement: Supplementary material 2 [file mgen-6-319-s002.pdf]

Supplemental Note and Figures for Yu et al. “Landscape of gene expression variation of natural isolates of *Cryptococcus neoformans* in response to biologically relevant stresses”

Supplemental Notes

Lineage specific DEG on mating-type loci (MAT)

Functional characterization of genes in the K-means clusters

Differential expression between clinical and environmental pairs

Supplementary references

Figure S1. Correlation matrix of all RNA-Seq samples.

Figure S2. Multi-dimensional scaling plots across lineages for each condition.

Figure S3. Number of lineage-specific DEGs identified in each condition and in the common set.

Figure S4. Percentage of GO terms for lineage DEGs in each condition.

Figure S5. Venn diagrams depicting overlap of DEGs between CSF and other conditions.

Supplemental Notes

Lineage-specific differentially expressed genes (DEGs) on mating-type loci (MAT)

To help understand if differences in gene expression between lineages correspond to gene presence/absence, we evaluated the presence of orthologs in Bt85 (VNBI) for each gene identified based on mapping to H99; 32 out of the 94 DEGs from the H99 comparison do not match an ortholog gene/group in Bt85 and were up-regulated in VNI strain. Most of these genes were located in five large regions, including Chromosome 1: 12789 - 19876 (CNAG_00002, CNAG_00003, and CNAG_00004), Chromosome 4: 1058232 - 1063107 (CNAG_07831, CNAG_05325, CNAG_05326), Chromosome 5: 7089 – 29713 (CNAG_06876, CNAG_06875, CNAG_06874, CNAG_06871, CNAG_06870, CNAG_06869), Chromosome 7: 384496 - 394513 (CNAG_06649, CNAG_06650, CNAG_06651, CNAG_06652, CNAG_06653), and Chromosome 8: 16125 - 32410 (CNAG_03086, CNAG_03087, CNAG_07707, CNAG_03090, CNAG_03092) **(Table S5c)**. H99 and Bt85 have different mating types, *MAT*α and *MAT***a**, respectively. As expected, three DEGs (CNAG_06812, CNAG_07015, and CNAG_07411) in the mating locus located on Chromosome 5 [1] were more highly expressed in H99 and down-regulated in Bt85 (**Table S5b**).

Functional characterization of genes in the K-means clusters

(1) Genes up-regulated during in vitro growth

The polysaccharide capsule is one of the main virulence factors of *C. neoformans* and plays a predominant role in the interaction with the host. This capsule, composed of glucuronoxylomannan and galactoxylomannan, not only provides resistance to stressful conditions but also has strong immunomodulatory properties to promote evasion and survival within the host [2, 3]. Many genes in k-means cluster 1 and cluster 5 are associated with cell wall and/or capsule formation (**Table S8a and S8e**). For instance, several genes are involved in the G protein-cAMP-PKA signaling pathway, which has been shown to promote mating and the production of virulence factors in *C. neoformans* [4, 5]. The *PKA1* gene

encodes the major cyclic AMP (cAMP)-dependent protein kinase catalytic subunit, and the *PKR1* gene encodes the protein kinase A (PKA) regulatory subunit. The low-affinity phosphodiesterase, Pde1, is involved in the PKA1-dependent regulation and modulates the cAMP level [6]. The *GPR4* gene encodes a G protein-coupled receptor that functions in sensing the amino acid methionine to activate cAMP-PKA signaling [7]. An adenylyl cyclase-associated protein encoded by the *ACA1* gene and the Gα subunit encoded by the *GPA1* gene cooperate to activate adenylyl cyclase, which plays a critical role in regulating melanin and capsule production, cell fusion, and filamentous growth [8]. Additional genes identified in this cluster include genes associated with acidification of vesicular compartments, growth at elevated temperatures, cell wall components, stress response, and capsule and melanin production. The vesicular (H+)-ATPase proton pump, encoded by the *VPH1* gene, is important for the acidification of vesicular compartments, and disruption of *VPH1* resulted in defective expression of polysaccharide capsule formation, growth at 37°C, and laccase and urease production [9]. The *FPD1*, *MP98*, and *CDA3* genes encode polysaccharide deacetylases, including chitin deacetylases. Chitin deacetylases deacetylate chitin, converting it to chitosan. Both chitin and chitosan are associated with the cell wall integrity of *C. neoformans* growing vegetatively [10] and are critical factors for the pathogenesis of *C. neoformans* [11–13]. In addition, *CSR1* encodes a chitin synthase regulator [13]. The Hex1 exochitinase, a hexosaminidase, degrades chitinoligomeric substrates to maintain cell wall plasticity [14]. Ssk1 and Ssk2 are involved in the stress-activated Pbs2-Hog1 mitogen-activated protein kinase (MAPK) signaling pathway, which governs several cellular events, including stress responses, sexual reproduction, drug sensitivity, and virulence [15]. The disruption of the *SSK2* gene in H99 enhanced capsule and melanin biosynthesis, mating efficiency, and hypersensitivity to stresses [16]. *KRE5* is a β-1,6-glucan synthesis-related gene that is vital for maintaining cell morphology and cell wall integrity and affects sensitivity to high temperature [17]. Other capsule or cell wall related genes are *EGCrP1* [18], *RIM20* [19], *WSP1* [20], *SCH9* [21], *GIB2*, *SSD1*, and *ISP3* [10]. The *MET3* gene encodes ATP sulfurylase and plays an important role in the methionine/cysteine biosynthetic pathway. Mutation of *MET3* has effects on capsule and melanin formation, as well as on

growth rate, thermotolerance, and virulence [22]. *LAC2* encodes a laccase important for melanin production and pathogenesis via Gα protein/cyclic AMP (cAMP) pathway [23]. Five other genes have also been found that when deleted are associated with melanization defects, including *RTF1*, *NUP75*, *CSN4*, *UBP14*, and *LAG1* [24].

Other potentially important virulence genes induced *in vitro* were also identified. *URE1* encodes the urease apoenzyme, which has been shown to have a role in virulence, especially during brain invasion [25, 26]. Ure1 regulates the expression of transporters and catabolic enzymes in response to the availability and type of nitrogen source in the environment [27]. Glr1 is a glutathione reductase which responds to nitric oxide stress [28]. The thioredoxin proteins, Trx1 and Trx2 are important for nitrosative stress resistance and virulence of *C. neoformans* [29]. In addition, we also found that more than 20 genes that encode V-type and F-type H+-transporting ATPases, which are ATP-driven enzymes using the energy of ATP hydrolysis to pump protons across membranes, including plasma and mitochondrial inner membranes. In fungi, preservation of a proton gradient across membranes is critical for various cellular metabolisms, including receptor-mediated endocytosis, intracellular membrane trafficking, pro-hormone processing, protein degradation, uptake of small molecules, and storage and detoxification of metabolites and ions [30]. *ATM1* encodes a mitochondrial ABC transporter that contributes to preservation of the homeostasis of cytosolic-nuclear Fe-S proteins during Cu stress [31].

(2) Genes up-regulated during *in vivo* growth

2 (a) Inositol-related proteins

We found 21 genes in the *in vivo* expression cluster are involved in inositol transport or catabolism, including myo-inositol transporters, inositol 2-dehydrogenases, inositol oxygenases, inositol/phosphatidylinositol kinases and phosphoinositide phospholipases (**Table S8c**). The human brain contains abundant inositol, which is a major osmolyte and plays a role in regulating normal neurological

responses [32]. If glucose availability is low in the brain, *Cryptococcus* may utilize inositol as a carbon source. Inositol in the brain has also been reported as a stimulator for promoting *Cryptococcus* penetration of the blood-brain barrier [33] and is required for virulence and mating [34, 35].

A large number of genes involved in DNA recombination, DNA repair, and DNA replication are also present in this cluster. These include DNA mismatch repair proteins (Pms2, Msh5, and Msh6), DNA polymerases, DNA primase, minichromosome maintenance proteins, DNA topoisomerases, exonucleases, endonucleases, and ATP-dependent DNA helicases. These results may reflect genome plasticity during the course of disease development within that host [36] and is already well-described with azole heteroresistance *in vivo* [2].

2 (b) Cell wall and capsule formation

A total of 120 known virulence-associated genes were also identified within this *in vivo* cluster. At least 47 of 120 virulence-associated genes identified in this cluster are involved in capsule formation or cell wall integrity (**Table S8c**). *UGE1* and *UGD1*, which encode UDP-glucose epimerase and UDP-glucose dehydrogenase, respectively, are necessary for galactoxylomannan biosynthesis and virulence in *C. neoformans* [3, 37]. In addition, several proteins, Skn1 and Kre62 - Kre64, are involved in the synthesis of polysaccharide β-1,6-glucan, which is a major component of the cell wall of *C. neoformans* [17]. Genes encoding capsule-associated proteins (Cap4; Cap5; Cap6; Cap10; Cap60; Cas32; Cas41; Cas42; Cas9; Gmt2) involved in capsule biosynthesis were highly expressed [38–40]. The chitin synthases, Chs1, Chs3 – Chs8, the chitin synthase regulators (Csr3), and the endochitinase, Chi21 are involved in chitin metabolic and biosynthetic processes [14, 41]. We also found two signaling components, a high affinity cAMP phosphodiesterase (Pde2) and a PKA catalytic subunit (Pka2), related to the cAMP/PKA pathway which promotes mating and the production of melanin and capsule. Pde2 may be regulated by Pka2, which contributes to the regulation of virulence. The deletion of the *PDE2* gene, however, has been shown to have modest effects on mating or melanin or capsule production [6].

2 (c) Cation transporters

Two active sodium (Na+) efflux transporters, Ena1 and Nha1, cooperatively maintain the intracellular pH and homeostasis of several toxic cations, such as Na+, Li+, and K+ [42] (**Table S8c**). Nha1, a Na+/H+ antiporter, is required for short-term adaptation to high salt shock. *ENA1* encodes a fungus-specific sodium or potassium P-type ATPase (Na(+)/ATPase), which is sensitive to alkaline pH conditions and essential for virulence in murine and rabbit models [43, 44]. Together, both genes play critical roles in cation homeostasis, pH regulation, membrane potential, and virulence in *C. neoformans* [42]. *PMC1* encodes a calcium transporter and provides tolerance to high Ca2+ concentrations. Pmc1 has been described as a vacuolar calcium ATPase, which enables *C. neoformans* to penetrate the central nervous system and is critical for both the progression of pulmonary infection and brain colonization in a murine infection model [45, 46].

2 (d) Stress responses

Genes involved in *C. neoformans* stress responses were also up-regulated *in vivo*. Sre1, a sterol regulatory element-binding protein (SREBP), functions in an oxygen-sensing pathway. This gene is involved in oxygen sensing and sterol homeostasis and is important for adaptation and growth of *C. neoformans* in the brain, which has suboptimal conditions of oxygen concentration and nutrition for fungal growth [47, 48] (**Table S8c**). In addition, three genes encoding MAP kinases, Bck1 (MAPKKK), Mkk2 (MAPKK), and the presumed terminal kinase Mpk1 (MAPK) comprising the Bck1-Mkk2-Mpk1 signaling pathway, which is activated in response to thermal stress, were up-regulated [49, 50]. We also found a pH-response transcription factor, Rim101, in this cluster. The Rim signal transduction pathway is well known to sense and respond to changes in host pH. Rim101, an alkaline-responsive transcription factor, is required for the proper formation of the protective polysaccharide capsule as well as growth under stressful host conditions, such as elevated cation concentrations and alkaline pH [19]. Lastly, the known virulence-associated gene,

CNA1, encoding the calcineurin A catalytic subunit, confers sensitivity to temperature, CO_2, and pH [51, 52].

2 (e) Other potential virulence factors

LIV5 encodes a protein homologous to one required for fungal pathogenesis and the deletion of this gene resulted had reduced virulence in a murine inhalation model [24].

(3) Genes up-regulated in CSF conditions, down-regulated in MP conditions

Many genes specifically up-regulated in CSF are tRNA/rRNA methyltransferases (Enzyme Commission (EC) number: 2.1.1.-) and tRNA pseudouridine synthases (EC 5.4.99.-). RNA methyltransferases catalyze the transfer of a methyl group on rRNA/tRNA and tRNA pseudouridine synthases catalyze the transition of uridine to pseudouridine on tRNA. In eukaryotes, these epigenetic modifications of rRNA nucleotides can alter ribosome function, which could allow modulation of translation and the cellular proteome in response to specific intracellular or environmental stresses [53, 54]. Six genes induced in CSF are linked to capsule formation, including *NSTX*, *CPS1*, *CRG1*, *GMT1*, *PBX1*, and *SET302* [10, 38, 55]. Also in this cluster are genes in the heat shock protein associated pathways. We identified several heat-shock related protein family members (Hsp60/Hsp70/Hsp90/Hsp104), including CNAG_00305, CNAG_06443, CNAG_05199, CNAG_03899, CNAG_01727, CNAG_01750, CNAG_03459, CNAG_05619, CNAG_07558, CNAG_00100, CNAG_06150, CNAG_06208, CNAG_07347, CNAG_01568, CNAG_03459, CNAG_05252, CNAG_03944, and CNAG_06106. Native misfolding of protein and protein aggregation caused by protein denaturation in pH, oxidative, osmotic, or high temperature stresses may lead to the loss of protein functions and may promote apoptosis suggesting that the CSF is a stressful environment [56].

Differential expression between clinical and environmental pairs

In comparing clinical and environmental isolates from VNI, we found 68 genes up-regulated and 137 genes down-regulated during MP interaction. Some of these genes were associated with fungal defense in macrophages and the survival in the host. For instance, we found the up-regulated gene, quinone oxidoreductase, which is a homolog of *S. cerevisiae ZTA1* gene (NADPH:quinone reductase). In *C. albicans*, quinone reductases promote resistance to oxidative stress and appear to be required for virulence in mouse infections studies [57]. The *SIT1* siderophore iron transporter (CNAG_00815), is up-regulated in clinical isolates, and is associated with iron acquisition, melanin formation, and cAMP signaling in *C. neoformans* [58]. In *Candida spp.*, the *SIT1* gene is required for epithelial invasion and penetration and for its siderophore-mediated iron acquisition that is critical for enhancing yeast survival to the microbicidal activities of macrophages [59, 60].

References

1. **Lengeler KB, Fox DS, Fraser JA, Allen A, Forrester K, *et al.*** Mating-type locus of *Cryptococcus neoformans*: a step in the evolution of sex chromosomes. *Eukaryot Cell* 2002;1:704–18.
2. **Sionov E, Chang YC, Kwon-Chung KJ**. Azole heteroresistance in *Cryptococcus neoformans*: emergence of resistant clones with chromosomal disomy in the mouse brain during fluconazole treatment. *Antimicrob Agents Chemother* 2013;57:5127–30.
3. **Moyrand F, Janbon G**. UGD1, encoding the *Cryptococcus neoformans* UDP-glucose dehydrogenase, is essential for growth at 37 degrees C and for capsule biosynthesis. *Eukaryot Cell* 2004;3:1601–8.
4. **D'Souza CA, Alspaugh JA, Yue C, Harashima T, Cox GM, *et al.*** Cyclic amp-dependent protein kinase controls virulence of the fungal pathogen *Cryptococcus neoformans*. *Mol Cell Biol* 2001;21:3179–3191.
5. **Alspaugh JA, Perfect JR, Heitman J**. *Cryptococcus neoformans* mating and virulence are regulated by the G-protein alpha subunit GPA1 and cAMP. *Genes Dev* 1997;11:3206–17.
6. **Hicks JK, Bahn Y-S, Heitman J**. Pde1 phosphodiesterase modulates cyclic AMP levels through a protein kinase A-mediated negative feedback loop in *Cryptococcus neoformans*. *Eukaryot Cell* 2005;4:1971–81.

7. **Xue C, Bahn Y-S, Cox GM, Heitman J**. G protein-coupled receptor Gpr4 senses amino acids and activates the cAMP-PKA pathway in *Cryptococcus neoformans*. *Mol Biol Cell* 2006;17:667–79.
8. **Bahn Y-S, Hicks JK, Giles SS, Cox GM, Heitman J**. Adenylyl cyclase-associated protein Aca1 regulates virulence and differentiation of *Cryptococcus neoformans* via the cyclic AMP-protein kinase A cascade. *Eukaryot Cell* 2004;3:1476–91.
9. **Erickson T, Liu L, Gueyikian A, Zhu X, Gibbons J, *et al.*** Multiple virulence factors of *Cryptococcus neoformans* are dependent on VPH1. *Mol Microbiol* 2001;42:1121–31.
10. **Donlin MJ, Upadhya R, Gerik KJ, Lam W, VanArendonk LG, *et al.*** Cross talk between the cell wall integrity and cyclic AMP/protein kinase a pathways in *Cryptococcus neoformans*. *MBio* 2014;5:e01573-14.
11. **Upadhya R, Baker LG, Lam WC, Specht CA, Donlin MJ, *et al.*** *Cryptococcus neoformans* Cda1 and its chitin deacetylase activity are required for fungal pathogenesis. *MBio* 2018;9:e02087-18.
12. **Baker LG, Specht CA, Donlin MJ, Lodge JK**. Chitosan, the deacetylated form of chitin, is necessary for cell wall integrity in *Cryptococcus neoformans*. *Eukaryot Cell* 2007;6:855–67.
13. **Baker LG, Specht CA, Lodge JK**. Cell wall chitosan is necessary for virulence in the opportunistic pathogen *Cryptococcus neoformans*. *Eukaryot Cell* 2011;10:1264–8.
14. **Baker LG, Specht CA, Lodge JK**. Chitinases are essential for sexual development but not vegetative growth in *Cryptococcus neoformans*. *Eukaryot Cell* 2009;8:1692–1705.
15. **Ko YJ, Yu YM, Kim GB, Lee GW, Maeng PJ, *et al.*** Remodeling of global transcription patterns of *Cryptococcus neoformans* genes mediated by the stress-activated HOG signaling pathways. *Eukaryot Cell* 2009;8:1197–1217.
16. **Bahn Y-S, Geunes-Boyer S, Heitman J**. Ssk2 mitogen-activated protein kinase kinase kinase governs divergent patterns of the stress-activated Hog1 signaling pathway in *Cryptococcus neoformans*. *Eukaryot Cell* 2007;6:2278–2289.
17. **Gilbert NM, Donlin MJ, Gerik KJ, Specht CA, Djordjevic JT, *et al.*** *KRE* genes are required for β-1,6-glucan synthesis, maintenance of capsule architecture and cell wall protein anchoring in *Cryptococcus neoformans*. *Mol Microbiol* 2010;76:517–534.
18. **Ishibashi Y, Ikeda K, Sakaguchi K, Okino N, Taguchi R, *et al.*** Quality control of fungus-specific glucosylceramide in *Cryptococcus neoformans* by endoglycoceramidase-related protein 1 (EGCrP1). *J Biol Chem* 2012;287:368–381.
19. **O'Meara TR, Norton D, Price MS, Hay C, Clements MF, *et al.*** Interaction of *Cryptococcus neoformans* Rim101 and protein kinase A regulates capsule. *PLoS Pathog* 2010;6:e1000776.

20. **Shen G, Whittington A, Wang P**. Wsp1, a GBD/CRIB domain-containing WASP homolog, is required for growth, morphogenesis, and virulence of *Cryptococcus neoformans*. *Eukaryot Cell* 2011;10:521–529.

21. **Wang P, Cox GM, Heitman J**. A Sch9 protein kinase homologue controlling virulence independently of the cAMP pathway in *Cryptococcus neoformans*. *Curr Genet* 2004;46:247–255.

22. **Yang Z, Pascon RC, McCusker JH, Alspaugh A, Cox GM**. Molecular and genetic analysis of the *Cryptococcus neoformans* MET3 gene and a met3 mutant a. *Microbiology* 2002;148:2617–2625.

23. **Pukkila-Worley R, Gerrald QD, Kraus PR, Boily M-J, Davis MJ, *et al.*** Transcriptional network of multiple capsule and melanin genes governed by the *Cryptococcus neoformans* cyclic AMP cascade. *Eukaryot Cell* 2005;4:190–201.

24. **Liu OW, Chun CD, Chow ED, Chen C, Madhani HD, *et al.*** Systematic genetic analysis of virulence in the human fungal pathogen *Cryptococcus neoformans*. *Cell* 2008;135:174–188.

25. **Cox GM, Mukherjee J, Cole GT, Casadevall A, Perfect JR**. Urease as a virulence factor in experimental cryptococcosis. *Infect Immun* 2000;68:443–8.

26. **Singh A, Panting RJ, Varma A, Saijo T, Waldron KJ, *et al.*** Factors required for activation of urease as a virulence determinant in *Cryptococcus neoformans*. *MBio* 2013;4:e00220-13.

27. **Marzluf GA**. Genetic regulation of nitrogen metabolism in the fungi. *Microbiol Mol Biol Rev* 1997;61:17–32.

28. **Missall TA, Pusateri ME, Donlin MJ, Chambers KT, Corbett JA, *et al.*** Posttranslational, translational, and transcriptional responses to nitric oxide stress in *Cryptococcus neoformans*: implications for virulence. *Eukaryot Cell* 2006;5:518–29.

29. **Missall TA, Lodge JK**. Function of the thioredoxin proteins in *Cryptococcus neoformans* during stress or virulence and regulation by putative transcriptional modulators. *Mol Microbiol* 2005;57:847–858.

30. **Veses V, Richards A, Gow NA**. Vacuoles and fungal biology. *Curr Opin Microbiol* 2008;11:503–510.

31. **Garcia-Santamarina S, Uzarska MA, Festa RA, Lill R, Thiele DJ**. *Cryptococcus neoformans* iron-sulfur protein biogenesis machinery is a novel layer of protection against cu stress. *MBio* 2017;8:e01742-17.

32. **Fisher SK, Novak JE, Agranoff BW**. Inositol and higher inositol phosphates in neural tissues: homeostasis, metabolism and functional significance. *J Neurochem* 2002;82:736–54.

33. **Liu T-B, Kim J-C, Wang Y, Toffaletti DL, Eugenin E, *et al.*** Brain inositol is a novel stimulator for promoting *Cryptococcus* penetration of the blood-brain barrier. *PLoS Pathog*

2013;9:e1003247.

34. **Xue C, Liu T, Chen L, Li W, Liu I, *et al.*** Role of an expanded inositol transporter repertoire in *Cryptococcus neoformans* sexual reproduction and virulence. *MBio* 2010;1:e00084-10.

35. **Xue C**. *Cryptococcus* and beyond—inositol utilization and its implications for the emergence of fungal virulence. *PLoS Pathog* 2012;8:e1002869.

36. **Boyce KJ, Wang Y, Verma S, Shakya VPS, Xue C, *et al.*** Mismatch repair of dna replication errors contributes to microevolution in the pathogenic fungus *Cryptococcus neoformans*. *MBio* 2017;8:e00595-17.

37. **Moyrand F, Lafontaine I, Fontaine T, Janbon G**. UGE1 and UGE2 regulate the UDP-glucose/UDP-galactose equilibrium in *Cryptococcus neoformans*. *Eukaryot Cell* 2008;7:2069–77.

38. **O'Meara TR, Andrew Alspaugh J**. The *Cryptococcus neoformans* capsule: A sword and a shield. *Clin Microbiol Rev* 2012;25:387–408.

39. **Okabayashi K, Hasegawa A, Watanabe T**. Microreview: Capsule-associated genes of *Cryptococcus neoformans*. *Mycopathologia* 2007;163:1–8.

40. **Moyrand F, Chang YC, Himmelreich U, Kwon-Chung KJ, Janbon G**. Cas3p belongs to a seven-member family of capsule structure designer proteins. *Eukaryot Cell* 2004;3:1513–24.

41. **Banks IR, Specht CA, Donlin MJ, Gerik KJ, Levitz SM, *et al.*** A chitin synthase and its regulator protein are critical for chitosan production and growth of the fungal pathogen *Cryptococcus neoformans*. *Eukaryot Cell* 2005;4:1902–12.

42. **Jung K-W, Strain AK, Nielsen K, Jung K-H, Bahn Y-S**. Two cation transporters Ena1 and Nha1 cooperatively modulate ion homeostasis, antifungal drug resistance, and virulence of *Cryptococcus neoformans* via the HOG pathway. *Fungal Genet Biol* 2012;49:332–345.

43. **Idnurm A, Walton FJ, Floyd A, Reedy JL, Heitman J**. Identification of ENA1 as a virulence gene of the human pathogenic fungus *Cryptococcus neoformans* through signature-tagged insertional mutagenesis. *Eukaryot Cell* 2009;8:315–26.

44. **Lee A, Toffaletti DL, Tenor J, Soderblom EJ, Thompson JW, *et al.*** Survival defects of *Cryptococcus neoformans* mutants exposed to human cerebrospinal fluid result in attenuated virulence in an experimental model of meningitis. *Infect Immun* 2010;78:4213–4225.

45. **Squizani ED, Oliveira NK, Reuwsaat JCV, Marques BM, Lopes W, *et al.*** Cryptococcal dissemination to the central nervous system requires the vacuolar calcium transporter Pmc1. *Cell Microbiol* 2018;20:e12803.

46. **Kmetzsch L, Staats CC, Cupertino JB, Fonseca FL, Rodrigues ML, *et al.*** The calcium transporter Pmc1 provides Ca^{2+} tolerance and influences the progression of murine cryptococcal infection. *FEBS J* 2013;280:4853–4864.

47. **Chang YC, Bien CM, Lee H, Espenshade PJ, Kwon-Chung KJ**. Sre1p, a regulator of oxygen sensing and sterol homeostasis, is required for virulence in *Cryptococcus neoformans*. *Mol Microbiol* 2007;64:614–29.
48. **Chang YC, Ingavale SS, Bien C, Espenshade P, Kwon-Chung KJ**. Conservation of the sterol regulatory element-binding protein pathway and its pathobiological importance in *Cryptococcus neoformans*. *Eukaryot Cell* 2009;8:1770–1779.
49. **Lam WC, Gerik KJ, Lodge JK**. Role of *Cryptococcus neoformans* Rho1 GTPases in the PKC1 signaling pathway in response to thermal stress. *Eukaryot Cell* 2013;12:118–131.
50. **Bahn Y-S, Jung K-W**. Stress signaling pathways for the pathogenicity of *Cryptococcus*. *Eukaryot Cell* 2013;12:1564–77.
51. **Odom A, Muir S, Lim E, Toffaletti DL, Perfect J, *et al.*** Calcineurin is required for virulence of *Cryptococcus neoformans*. *EMBO J* 1997;16:2576–2589.
52. **Cruz MC, Sia RA, Olson M, Cox GM, Heitman J**. Comparison of the roles of calcineurin in physiology and virulence in serotype D and serotype A strains of *Cryptococcus neoformans*. *Infect Immun* 2000;68:982–5.
53. **Sloan KE, Warda AS, Sharma S, Entian K-D, Lafontaine DLJ, *et al.*** Tuning the ribosome: The influence of rRNA modification on eukaryotic ribosome biogenesis and function. *RNA Biol* 2017;14:1138–1152.
54. **Chan CTY, Dyavaiah M, DeMott MS, Taghizadeh K, Dedon PC, *et al.*** A quantitative systems approach reveals dynamic control of trna modifications during cellular stress. *PLoS Genet* 2010;6:e1001247.
55. **Maier EJ, Haynes BC, Gish SR, Wang ZA, Skowyra ML, *et al.*** Model-driven mapping of transcriptional networks reveals the circuitry and dynamics of virulence regulation. *Genome Res* 2015;125:690–700.
56. **Tiwari S, Thakur R, Shankar J**. Role of heat-shock proteins in cellular function and in the biology of fungi. *Biotechnol Res Int* 2015;2015:132635.
57. **Li L, Naseem S, Sharma S, Konopka JB**. Flavodoxin-like proteins protect *Candida albicans* from oxidative stress and promote virulence. *PLOS Pathog* 2015;11:e1005147.
58. **Tangen KL, Jung WH, Sham AP, Lian T, Kronstad JW**. The iron- and cAMP-regulated gene SIT1 influences ferrioxamine B utilization, melanization and cell wall structure in *Cryptococcus neoformans*. *Microbiology* 2007;153:29–41.
59. **Heymann P, Gerads M, Schaller M, Dromer F, Winkelmann G, *et al.*** The siderophore iron transporter of *Candida albicans* (Sit1p/Arn1p) mediates uptake of ferrichrome-type siderophores and is required for epithelial invasion. *Infect Immun* 2002;70:5246–55.

60. **Nevitt T, Thiele DJ**. Host iron withholding demands siderophore utilization for *Candida glabrata* to survive macrophage killing. *PLoS Pathog* 2011;7:e1001322.

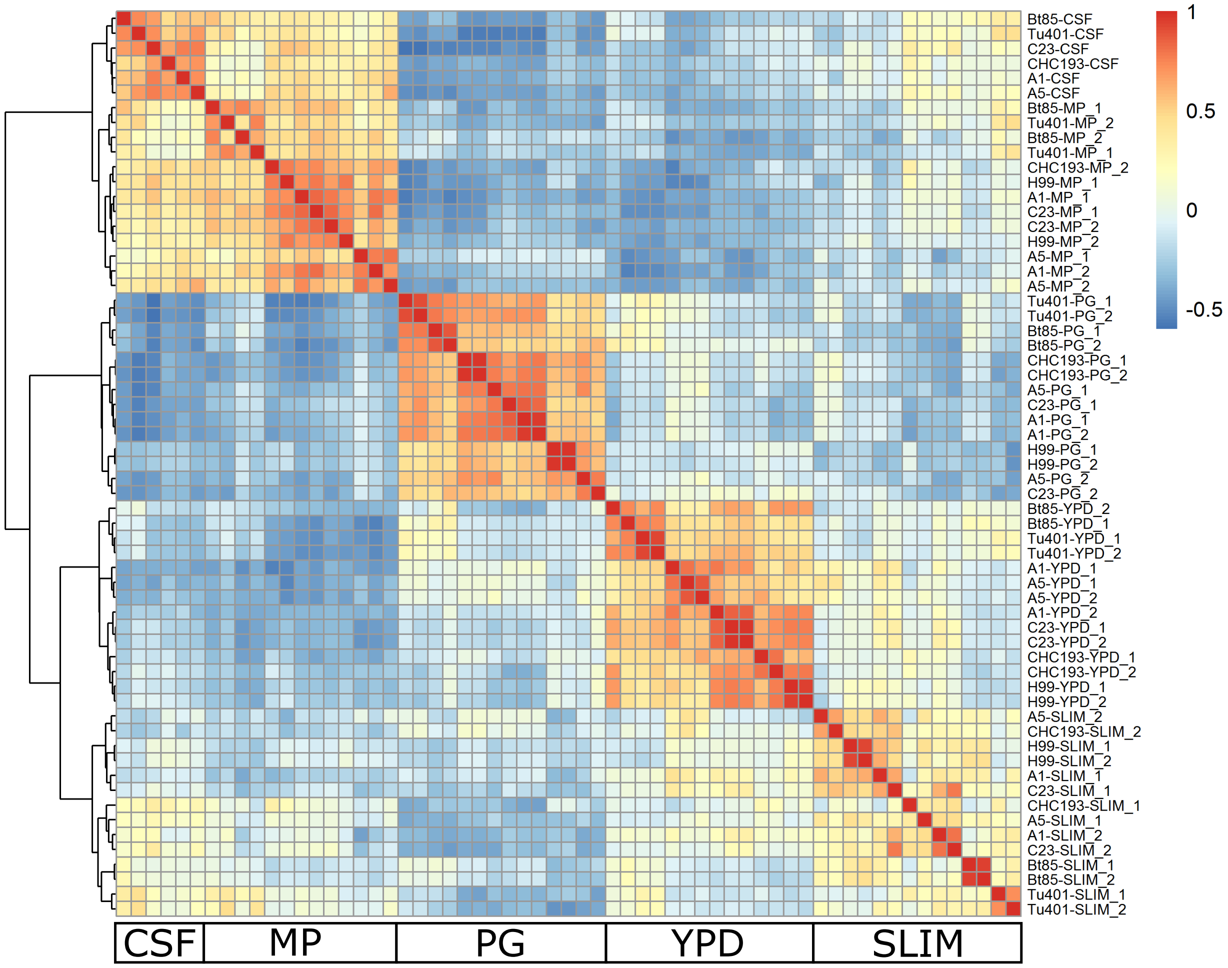

Figure S1. Correlation matrix of all RNA-Seq samples. Sample correlation matrix displays the overall similarity in expression profile for each sample from different conditions. Red color intensity indicates increasing sample correlation, whereas blue color intensity indicates decreasing sample correlation. The dendrogram clustering on the Y axis indicates the overall similarity of all samples. The samples are named with the strain name, the condition, and the experimental replicate.

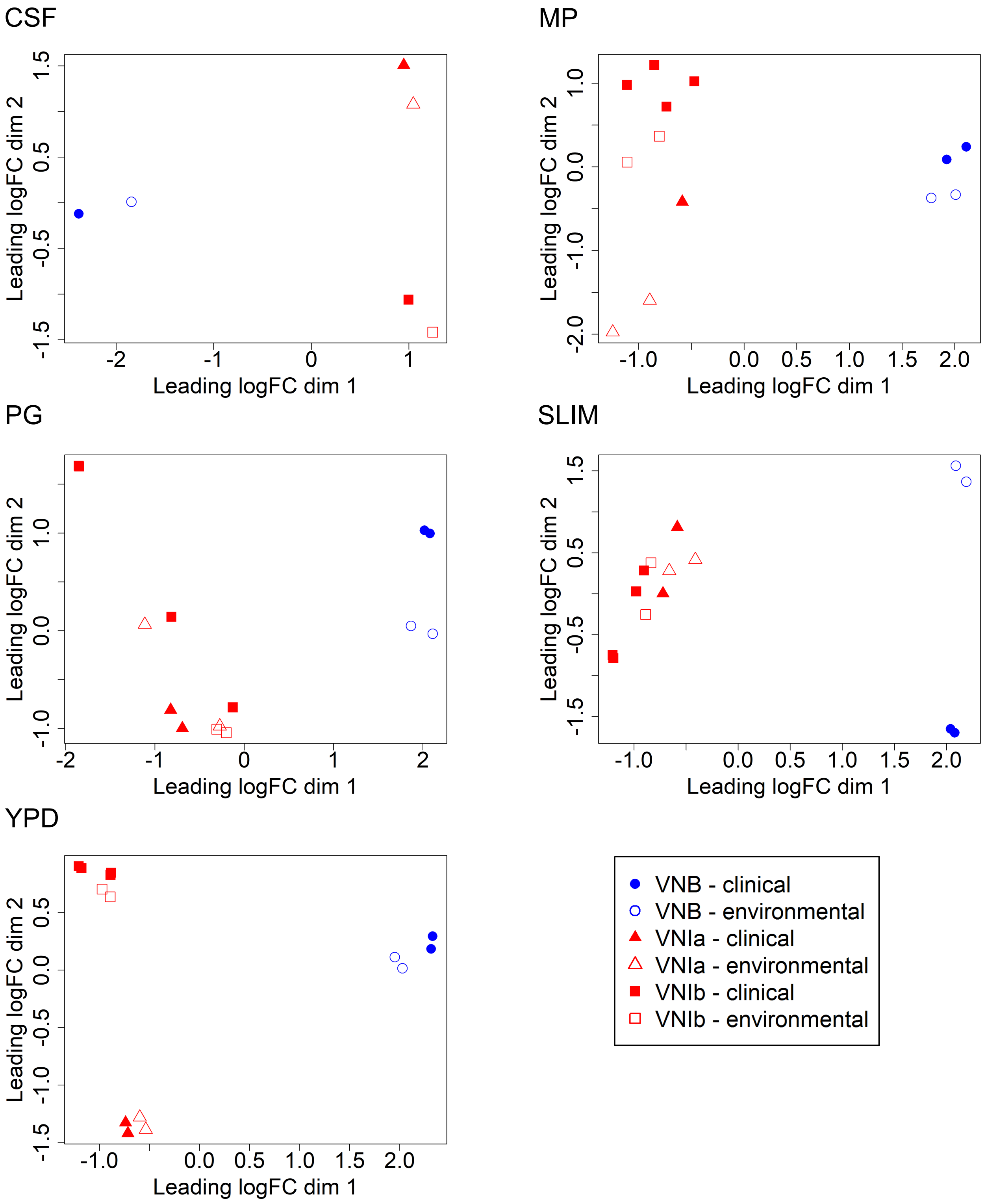

Figure S2. Multi-dimensional scaling plots across lineages for each condition. These MDS plots show the clear separation among two lineages, VNB and VNI (including sublineages VNIa and VNIb), for each condition, including VNIa clinical (filled red triangle) and environmental (open red triangle) isolates; VNIb clinical (filled red square) and environmental (open red square) isolates; VNB clinical (filled blue circle) and environmental (open blue circle) isolates.

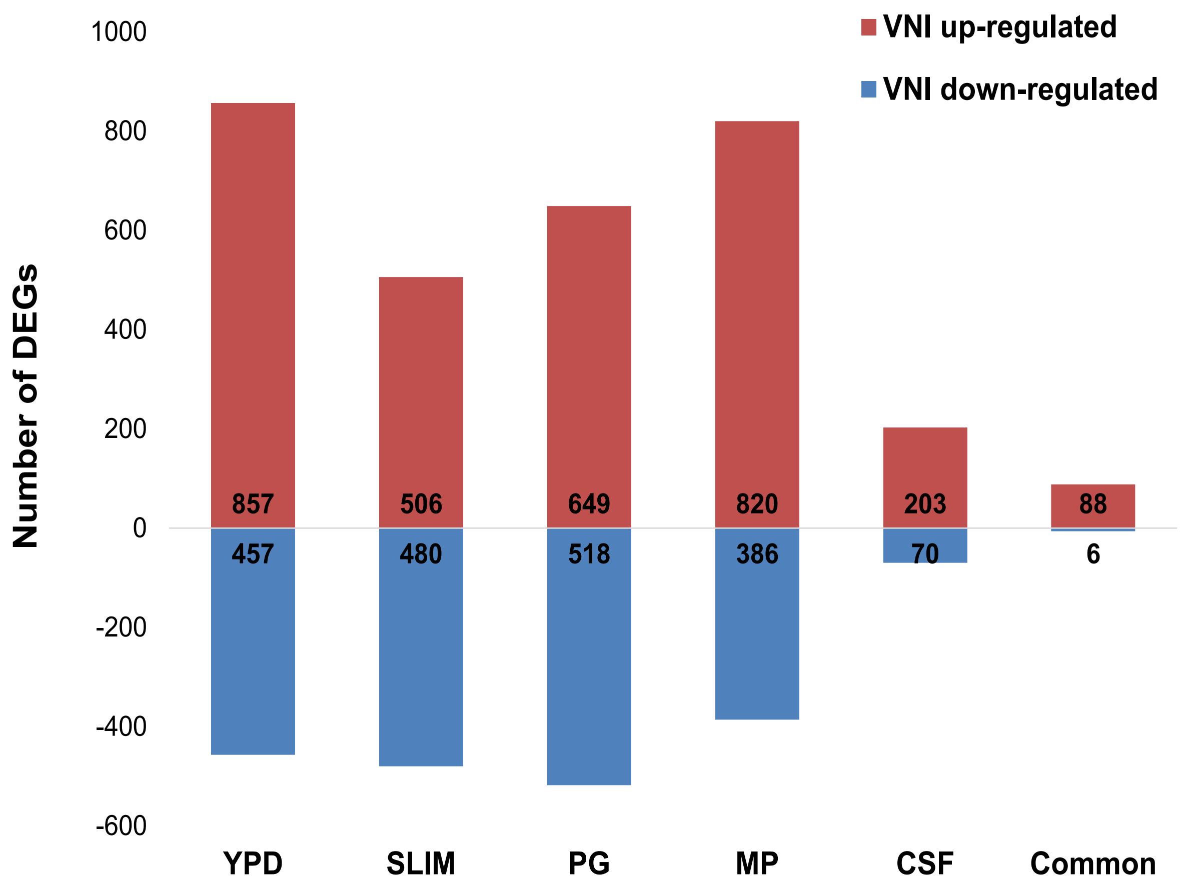

Figure S3. Number of lineage-specific differential expressed genes (DEGs) identified in each condition and in the common set. The significant differential expression genes are identified between VNI and VNB lineages for each condition by exact test at FDR *P*-value < 0.05. The red bar represents the number of DEGs that are up-regulated in VNI strains, compared to VNB strains. By contrast, the blue bar represents the number of VNI down-regulated DEGs. The common category is the intersection of lineage specific DEGs across all five conditions (94 genes).

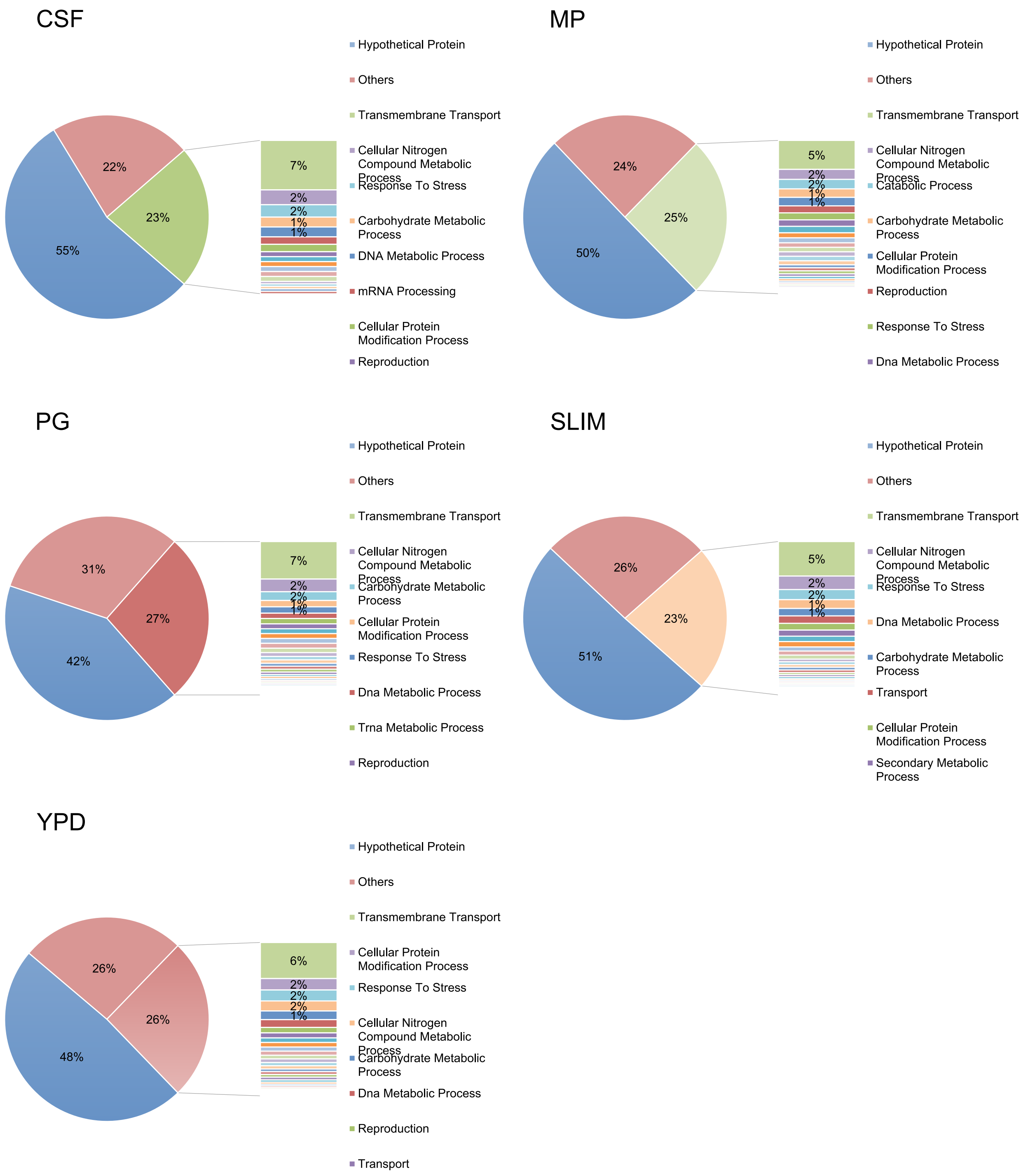

Figure S4. Percentage of GO terms for lineage DEGs in each condition. The GO SLIM terms of DEGs were mapped by the FungiDB database. Genes without GO SLIM annotations were classified in two categories; Hypothetical Proteins do not have assigned names or annotated functions in FungiDb and Others have an assigned name or function although not an assigned GO SLIM term.

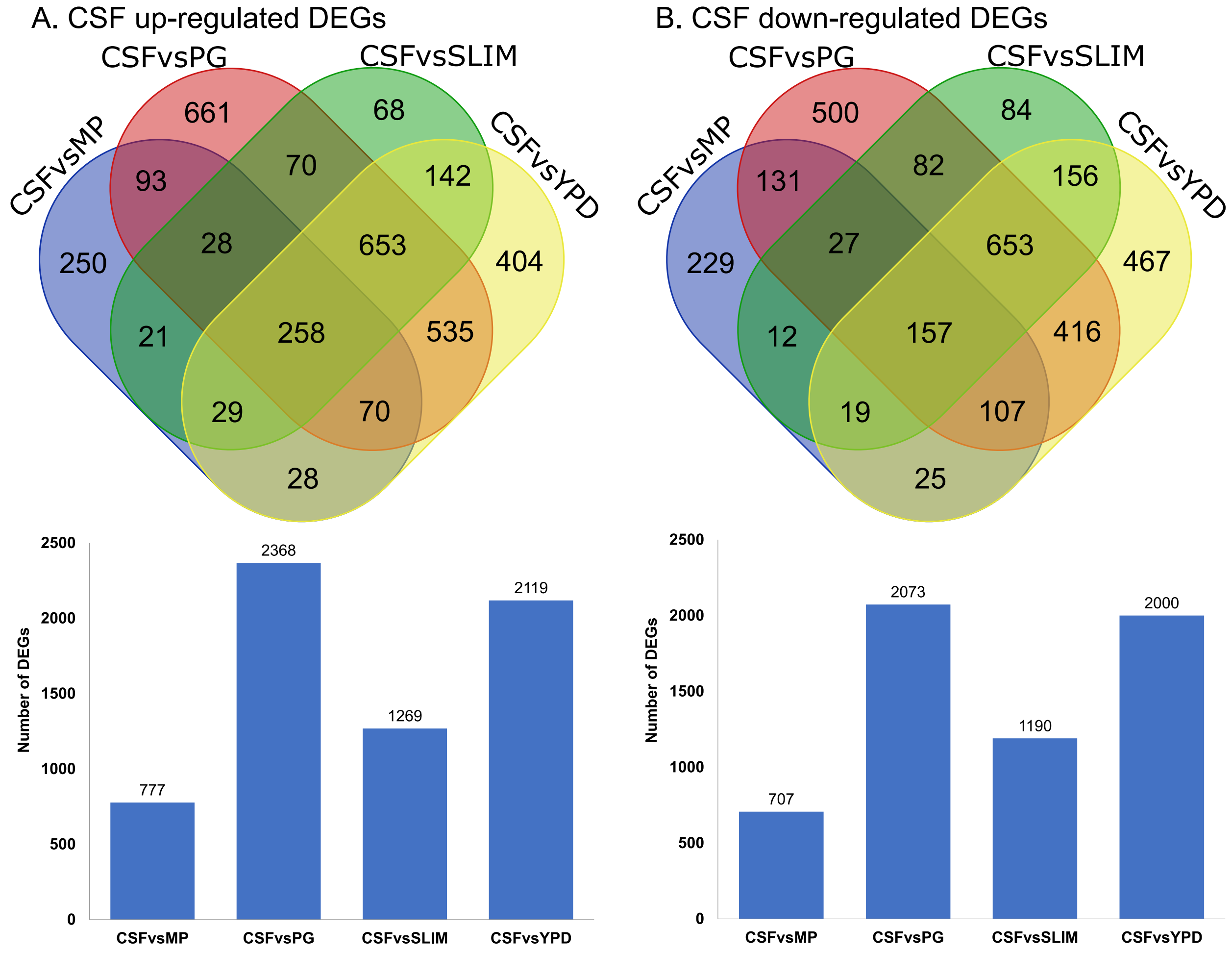

Figure S5. Venn diagrams depicting the overlap of DEGs between CSF and other conditions. The plots show the number of DEGs between CSF and each of the other conditions and the overlaps between these DEG sets. A. Up-regulated DEGs in CSF condition compared to the other conditions. B. Down-regulated DEGs in CSF condition compared to the other conditions. The total number of up-regulated (lower left panel) and down-regulated CSF (lower right panel) compared to the other conditions varies depending on the condition.
